# Energy requirements of growing small ruminants raised for meat production in contrasting climatic regions: a meta-analysis

**DOI:** 10.1093/tas/txaf012

**Published:** 2025-02-05

**Authors:** Vinícius C Souza, Adeline Bougouin, Harry Archimede, Adegbola Adesogan, Ermias Kebreab

**Affiliations:** Department of Animal Science, University of California, Davis, CA 95616, USA; Department of Animal Science, University of California, Davis, CA 95616, USA; INRAE, Unité de Recherches Zootechniques, 97170 Petit-Bourg, France; Department of Animal Sciences, University of Florida, Gainesville, FL 32611, USA; Department of Animal Science, University of California, Davis, CA 95616, USA

**Keywords:** diet formulation, goat, sheep, tropics

## Abstract

The objective of this meta-regression was to evaluate the influence of ruminant species, sex, and climatic regions on the metabolizable energy (**ME**) requirements for maintenance (**MEm)** and weight gain (**MEg**) in growing small ruminants raised for meat production across different climatic regions. Data included 655 and 337 treatment means from 173 and 99 studies on sheep and goats, respectively. Metabolizable energy intake (**MEI**; MJ/kg^^0.75^) was regressed against average daily gain (**ADG**; g/kg^^0.75^), with the study included as a random effect. The analysis found that MEm was not affected by species (*P* = 0.50), but MEg (MJ/g ADG) was significantly different between species (*P* = 0.02), with sheep requiring 0.032 (± 0.002) and goats 0.026 (± 0.002) MJ/g ADG. Sex did not affect MEm in either species (*P* ≥ 0.32). However, in goats, intact males had a greater MEg (*P* = 0.02) than females (0.030 ± 0.003 vs. 0.013 ± 0.006 MJ/g ADG). MEm was lower (*P* = 0.03) in small ruminants raised in subtropical regions (0.497 ± 0.046 MJ/kg^^0.75^) compared to those in semi-arid (0.600 ± 0.038 MJ/kg^^0.75^) and tended to be lower than those in arid regions (0.529 ± 0.050 MJ/kg^^0.75^). However, these differences disappeared when adjusting for maturity, diet composition, digestibility, or altitude. MEg was significantly higher (P < 0.05) in animals raised in arid regions (0.032 ± 0.006 MJ/g ADG) compared to those in Mediterranean (0.009 ± 0.004 MJ/g ADG) or semi-arid regions (0.009 ± 0.004 MJ/g ADG) after adjusting for diet composition and digestibility. Similarly, ruminants in Mediterranean regions had lower MEg (0.019 ± 0.004 and 0.009 ± 0.004 MJ/g ADG) than those in tropical regions (0.033 ± 0.002 and 0.024 ± 0.002 MJ/g ADG), respectively after adjusting for maturity, diet composition, and digestibility. MEg in semi-arid regions was consistently lower than in tropical regions, regardless of the covariates tested. For predictive purposes, the global model exhibited the best accuracy (CCC = 0.57 and RSR = 0.79), comparable to the model derived specifically for the tropical region (CCC = 0.58 and RSR = 0.80). This meta-analysis provides a comprehensive evaluation of species-specific differences in ME requirements in small ruminants while recognizing the challenges posed by confounding effects and climatic variability inherent in global datasets. The analysis suggests that animals raised in tropical conditions may have lower MEm than current feeding systems that use data from temperate climates.

## Teaser

Small ruminants raised in tropical conditions may have lower energy requirements for maintenance than recommend by current feeding systems, especially those developed primarily using data obtained in temperate climates. The global and tropical models developed in this study are recommended for use in ration formulation software, being the later targeted towards small ruminants in tropical regions.

## INTRODUCTION

Sheep and goat production in the tropics is of economic importance as small ruminants have the ability to convert forage and byproducts (i.e., non-human edible food) into milk, meat, and fiber. Most of the feeding systems, such as the Agricultural and Food Research Council (AFRC, 1993), Agricultural Research Council (ARC, 1984), and the Institut Nationale de la Recherche Agronomique (INRA, 2018) are based on nutrient requirements of ruminants raised in temperate climates, thus are not fully appliable to tropical conditions. Farming systems in temperate and tropical countries differ in climatic conditions, nutritional values of diets, and animal genotypes. The most common breeds used in tropical environments are crossbreeds, which have genotypes suited to be both highly productive and adapted to the local conditions. Adaptation to diet and climatic conditions affects nutrient partitioning, animal growth, body composition, and, consequently, energy and protein requirements (NRC, 2007). However, comparisons of energy requirements for small ruminants raised in different climatic regions are scarce. Salah et al. (2014) performed a meta-analysis on energy and protein requirements of tropical small ruminants and reported that both energy and protein requirements for maintenance are greater than those raised in temperate countries. Several calorimetric studies showed that net energy (**NE**) requirement for maintenance (**NEm**) for sheep raised in different climatic conditions were greater than those recommended by National Research Council (Deng et al., 2014; Salah et al., 2014; Rodrigues et al., 2016; Yang et al., 2020). In another study, Dawson and Steen (1998) using data from multiple calorimetry experiments in the United Kingdom showed that the metabolizable energy (**ME**) requirement for maintenance (**MEm**) for growing lambs was much higher than that proposed by AFRC (1993). This information shows the importance of calculating more accurate values for local breeds raised in different climate scenarios. More accurate values for the energy requirement for maintenance and growth could lead to better-suited diets, which would help to increase animal productivity, particularly in tropical regions.

The estimation of energy requirements for small ruminants has evolved over the past decades, with increasing emphasis on methods that account for NE requirements and their efficiencies (Teixeira et al., 2024). While the dose-response method, as utilized in the NRC (2007) for small ruminants, remains a practical and widely applied approach, more recent efforts, such as the BR-Caprinos & Ovinos system (BR-Caprinos & Ovinos, 2024), have focused on refining energy requirement models on a net energy basis to better account for variations in environmental conditions and production systems. However, due to the challenges of addressing climatic differences worldwide in a single study and the lack of comprehensive datasets on NE requirements across various climatic regions, the use of the dose-response method is still useful. These challenges underscore the importance of developing region- and species-specific energy requirement estimates. Additionally, meta-analysis techniques can offer a broader understanding of how climatic regions influence the metabolic energy requirements in small ruminants and serve as a strong tool for synthesizing diverse data sources.

Regression analysis of feeding trial data can provide estimates of nutrient requirements for animals kept under certain conditions. Salah et al. (2014) conducted a meta-analysis to evaluate the energy requirements of ruminants in tropical regions. However, the authors have not evaluated the impacts of specific climatic regions on MEm and metabolizable energy requirements for weight gain (**MEg**). Thus, the objectives of this study were to: 1) collate and update a large database of independent feeding trial studies in different climate scenarios, 2) run a meta-analysis taking into account the maximum diversity of animal genotypes and dietary systems to compare the effects of ruminant species (sheep and goat), sex (male, female, and male castrated), and climatic regions (tropical, subtropical, arid, semi-arid, and Mediterranean) on MEm and MEg, and 3) to provide estimates of energy requirements of growing small ruminants for meat production specific to each climatic region.

## MATERIALS AND METHODS

Animal Care and Use Committee approval was not required for this study because the data were obtained from published literature.

### Data Collection

The database was created based on searches for pertinent studies in Science Direct and CAB international databases and by reporting treatment mean measurements and their respective number of observations from independent feeding studies. The literature search was completed with the objective of estimating energy requirements for maintenance and gain for growing small ruminants in tropical and subtropical climates. Thus, the keywords used in the search included “sheep” or “goats,” “feeding trial,” “tropics,” and “requirement.” The search refinement criteria were kept relatively broad so that all possible studies could be located and included. Search refinement criteria included the language English and French, the location (regions that lie between the latitude lines of the tropics and subtropical zones), and published after 2013 until 2019 in order to update the database used in Salah et al. (2014).

The search initially resulted in over 800 and 300 papers identified for sheep and goats, respectively (Figures S1 and S2) through database searching. For inclusion in the database, experiments must have reported the following criteria: 1) either dietary ingredients or measurements of dietary contents; 2) measurements of daily intakes, ideally dry matter intake (**DMI**) and metabolizable energy intake (**MEI**); 3) measurements of performances [average daily gain (**ADG**), initial and final body weight (**BW**)]; and 4) characteristics of individual animals (breed, age). Measurements of energy intakes were collected as well, if available. The PRISMA 2020 scheme of the search and selection process is given in Figure S1 for sheep and Figure S2 for goats (Page et al., 2021). A total of 116 observations from 24 studies in sheep and 8 new studies met the criteria adopted and were included.

**Figure 1.**
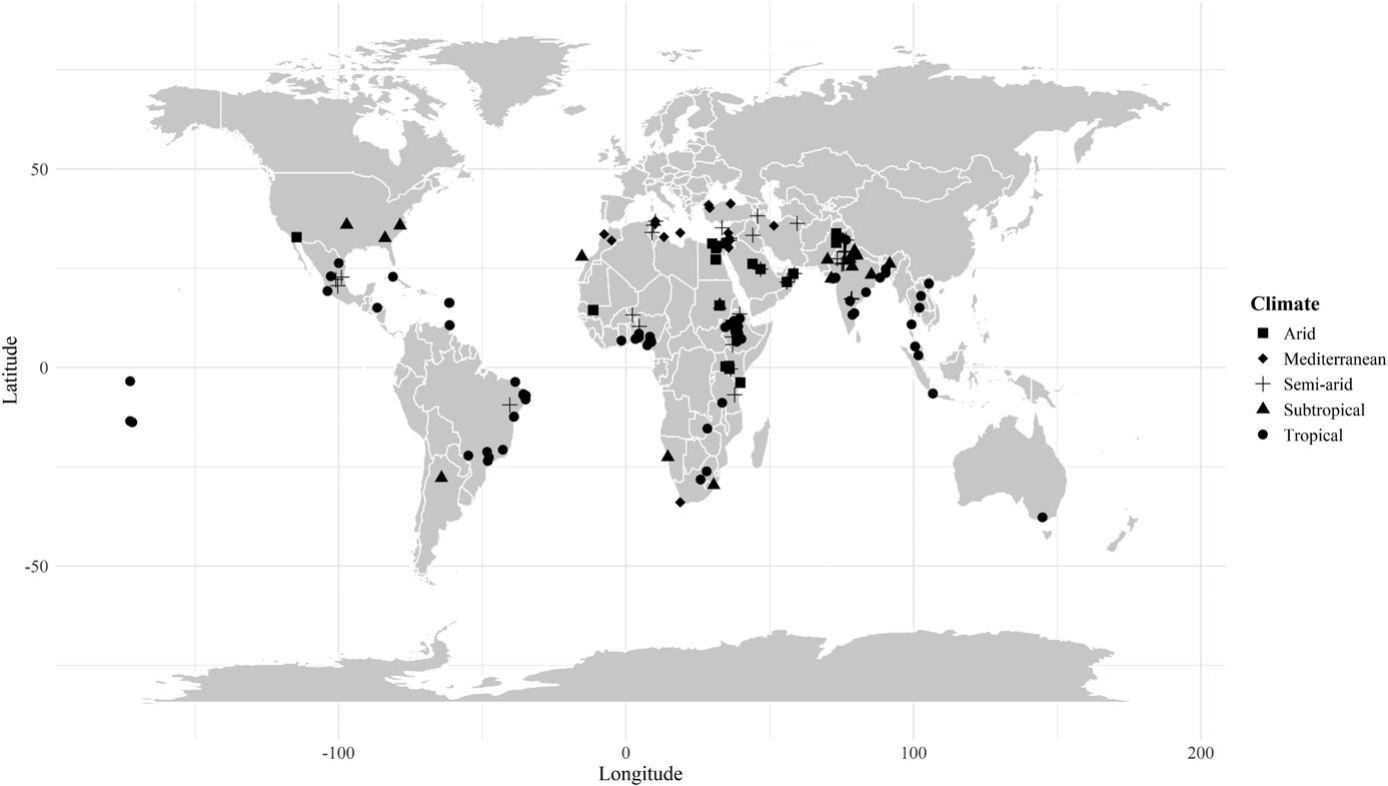
Global distribution of studies (n = 272) used for model development across climatic regions. Arid = 25 studies, Mediterranean = 39 studies, Semi-arid = 55 studies, Subtropical = 39 studies, and Tropical = 114 studies.

The initial database included 1,069 observations from 175 and 101 studies conducted in various climatic conditions in growing sheep and goats in our analysis, respectively. From the initial database, a total of 953 observations from 151 and 93 studies on sheep and goats were used in the study of Salah et al. (2014), respectively. We have only used studies from countries within the tropical and subtropical zones from the Salah et al. (2014) database. The final database, after outlier removal, included 655 and 337 treatment means from 173 and 99 studies from sheep and goats, respectively. These were further classified into five climate regions according to their geographical location: tropical, subtropical, arid, semi-arid, and Mediterranean (Figure 1). A list of the studies included in the database can be found in Tables S1 and S2.

### Animals and Diets Used in the Feeding Experiments

The sheep diets were diverse, the majority being mixed diets (95%) and the rest being exclusively forage-based diets (5%). Diets were based on several different types of forage such as hay (37%), straw (26%), pasture (9%), and others (plant part, tree leaves, pulps, silage; 23%). In a few studies, the forage form was not specified (2.6%). Wheat straw was the most commonly used straw, while alfalfa and Tifton-85 hays represent the majority of hay types. Concentrates were generally composed of conventional ingredients, such as wheat, corn, or barley grains, as well as soybean meal, although unconventional resources were also used, such as olive cake meals, cottonseed meals, castor bean meals, or sesame meals.

For the goat dataset, diets were for the most part based on hay and straw (37% and 33%, respectively). Similarly, for the sheep dataset, common concentrates were used in the studies, such as barley, corn or wheat grains, and soybean meal while groundnut cake or cottonseed meal were also fed to goats.

### Estimations and Calculations

The most important parameters considered were ADG (g/d), BW (kg), DMI (g/d), ME concentration (MJ/kg DM), MEI (MJ/d), sexual condition (intact male, female, castrated male), ruminant species (goat or sheep), and climate conditions according to the geographic location where the trials were conducted. When MEI was not reported in the publication but ME concentration was reported, we calculated MEI by multiplying ME concentration by the respective DMI. In studies where neither ME concentration nor MEI were reported, we estimated MEI following one of the approaches described below in Equations 1-4:

1) Predicted MEI using the equation described in the AFRC (1993):

$$\begin{array}{l} MEI\;\left(MJ/d\right)=digestible\;organic\;matter\;intake\;\left(g/d\right)\times .0157 \end{array}$$ (1)

2) Predicted MEI from total digestible nutrients (**TDN**; % DM) as described in the NRC (2001):

$$\begin{array}{l} Digestible\;energy\left(DE,\;MJ/kg\right)=0.044\times TDN\;\left(\%\;DM\right)\times 4.18 \end{array}$$ (2)

$$\begin{array}{l} ME\;\left(MJ/kg\right)=1.01\times DE\;\left(MJ/kg\right)--1.88 \end{array}$$ (3)

$$\begin{array}{l} MEI\;\left(MJ/d\right)=\left[DMI\;\left(g/d\right)\times ME\;\left(MJ/kg\right)\right]÷1000 \end{array}$$ (4)

The MEI was estimated from TDN only in few instances, where digestible organic matter intake was not reported or could not be calculated from organic matter intake and digestibility. Net energy intake or concentration was not reported in the majority of the studies included in our dataset. Thus, our focus in this meta-analysis was on ME requirements.

Because of the variability in BW within and between ruminant species, we standardized the variables of interest such as ADG and MEI by expressing them per unit of metabolic BW (kg^0.75^) (Kleiber, 1975). For instance, MEI per kg of BW^0.75^ and ADG per kg of BW^0.75^ were used. The metabolic BW was calculated by averaging the initial and final BW and raising it to the power of 0.75.

### Statistical Analyses

All data were analyzed using R version 4.1.1 (R Core Team, 2021). The dataset was analyzed for outliers by first visually checking boxplots in R (boxplot function, version 0.98.1102) and then using the interquartile range (**IQR**) method (Zwillinger and Kokoska, 2000). A factor of 1.5 for extremes was used in constructing markers to identify outliers, as shown in Equations 5-7:


$$\begin{array}{l} IQR\;=\;Third\;Quartile\;\left(Q3\right)\;--\;First\;Quartile\;\left(Q1\right) \end{array}$$ (5)

$$\begin{array}{l} Lower\;Fence\;=\;Q1\;\text{-}\;IQR\;\times \;1.5 \end{array}$$ (6)

$$\begin{array}{l} Upper\;Fence\;=\;Q3\;+\;IQR\;x\;1.5 \end{array}$$ (7)

A meta-regression analysis was conducted to quantify the relationship between MEI (MJ/kg^0.75^) and ADG (g/kg^0.75^) and how this relationship is affected by species, sex, and climatic regions. In order to evaluate the benefit of developing models predicting ME requirements specific to each region and sex, we created 9 subsets (3 sexes and 6 climate regions) of data using the subset function from R, and derived models predicting ME requirements specific for each subset. Models were derived using the R package lme4 version 3.4.3 (Bates et al., 2015) for linear mixed-effects regression with a random intercept for the study effect (St-Pierre, 2001). Error normality was checked at each step of the model derivation process through evaluation of residual plots. The weight used in the meta-regression was calculated by the square root of the number of observations of each study as performed by Hanigan et al. (2021). We used the “relevel” function to change reference level used in the factor variables such as ruminant species, sex, and climate region for the intercepts and slopes, and then we applied a *t*-test to determine whether the intercepts and slopes within ruminant species, sexes, and climate conditions differed statistically. For instance, for the factor variable sex the reference level was castrated male, thus the intercept and slopes reported for female and male were the calculated difference from their actual intercepts and slopes and the intercept and slopes reported for castrated male. However, such models did not allow us to evaluate whether or not the slopes and intercepts differ between male and female. By applying the relevel function, the reference level can be changed from castrated male to male and thus compare intercepts and slope between males and females. For the purpose of this meta-analysis, the different intercepts were assumed to be the MEm (MJ/kg^0.75^) and the slopes denoted the MEg (MJ/g ADG) (Luo et al., 2004).

### Model Evaluation

After derivation by the above procedure, a Monte Carlo cross-evaluation was performed to evaluate the predictive ability of fitted prediction models as described by Souza and White (2021). Briefly, the data set was randomly divided where 60% of the treatments were used for model derivation and 40% of the data were used for model evaluation. The data splitting, model derivation, and model evaluation were repeated 100 times using unique strings of random numbers to identify which observations were used for evaluation and which were used for evaluation. Model quality was assessed on the basis of the root mean square error (**RMSE**), expressed as an absolute value or percentage (**RMSPE**) of observed MEI (g/kg^0,75^), RMSE-observations standard deviation ratio (**RSR**), and concordance correlation coefficient (**CCC**). The mean squared prediction error was decomposed into mean (**MB**) and slope bias (**SB**) deviations to identify systematic biases (Bougouin et al., 2022).

The ratio of RMSPE and standard deviation of the data (observed values), namely RSR can be used to compare the performance of models developed from different datasets. This approach considers standardized model performance relative to the variability in observations from different datasets (Moriasi et al., 2007). Smaller RSR (<1) indicates superior performance given the variability of observations (Bougouin et al., 2022). Lin’s concordance correlation coefficient was used to evaluate the agreement between variables (Lin, 1989). Coefficients were obtained using predicted values that were calibrated for study effects. For final comparisons of models using different derivation data sets, CCC was the main tool used for model comparison.

## RESULTS

### Dataset

Before conducting the outlier analysis, we excluded 44 observations from the initial database due to their negative ADG values. This step was taken to ensure that only data from growing animals was used for model development. During the outlier analysis, 33 MEI observations that were considered outliers were identified and removed (Figure S3), which corresponded to 3.22% of the data available for model development. Descriptive statistics of the variables used for model development after outlier removal are presented in Table 1. The average MEI was 0.77 (± 0.24) and 0.69 (± 0.23) MJ/kg BW^0.75^ for sheep and goats, respectively. Average daily gain was 10.2 (± 5.71) and 7.30 (± 4.86) g/kg^0.75^ for sheep and goats, respectively.

**Table 1.** Summary statistics of variables used for model development

Variables^1^	n^2^	Mean	SD	Minimum	Maximum
**General**					
ME intake (MJ/d)	992	7.81	3.89	0.580	26.4
ADG (g/d)	992	100	76.2	0.22	374
Average BW (kg)	992	22.5	8.31	6.37	55.6
MBW (kg^0.75^)	992	10.2	2.83	4.01	20.4
ME intake (MJ/BW^0.75^)	992	0.74	0.24	0.11	1.44
ADG (g/kg BW^0.75^)	992	9.20	5.60	0.02	36.6
SRW (kg)	850	48.5	20.2	25	113
Z	850	0.50	0.18	0.10	1.07
Altitude (m)	961	720	794	−230	3,000
**Sheep**					
ME intake (MJ/d)	655	8.76	4.08	1.47	26.4
ADG (g/d)	655	118	80.6	0.22	374
Average BW (kg)	655	24.7	7.93	8.42	55.6
MBW (kg^0.75^)	655	11.0	2.63	4.94	20.4
ME intake (MJ/BW^0.75^)	655	0.77	0.24	0.21	1.44
ADG (g/kg BW^0.75^)	655	10.2	5.71	0.02	31.9
SRW (kg)	535	50.4	20.0	25.0	113
Z	535	0.54	0.17	0.20	1.07
Altitude (m)	635	834	839	−230	3,000
**Goats**					
ME intake (MJ/d)	337	5.96	2.64	0.58	15.0
ADG (g/d)	337	64.2	50.1	2.90	326
Average BW (kg)	337	18.1	7.27	6.37	41.4
MBW (kg^0.75^)	337	8.67	2.59	4.01	16.3
ME intake (MJ/BW^0.75^)	337	0.69	0.23	0.11	1.42
ADG (g/kg BW^0.75^)	337	7.30	4.86	0.35	36.6
SRW (kg)	315	45.3	20.2	25.0	113
Z	315	0.43	0.17	0.10	1.00
Altitude (m)	326	507	651	3	2,325
**Tropical region**					
ME intake (MJ/d)	477	6.40	2.88	1.47	17.2
ADG (g/d)	477	76.6	61.1	0.22	281
Average BW (kg)	477	19.3	5.81	6.37	32.4
MBW (kg^0.75^)	477	9.12	2.10	4.01	13.6
ME intake (MJ/BW^0.75^)	477	0.69	0.22	0.21	1.44
ADG (g/kg BW^0.75^)	477	7.99	5.18	0.02	36.6
SRW (kg)	410	42.3	17.6	25	100
Z	410	0.49	0.18	0.10	1.07
Altitude (m)	464	880	902	5.00	2,890
**Subtropical region**					
ME intake (MJ/d)	124	5.85	2.04	2.27	11.2
ADG (g/d)	124	72.0	44.3	5.60	204
Average BW (kg)	124	20.4	9.14	7.35	41.4
MBW (kg^0.75^)	124	9.42	3.17	4.46	16.3
ME intake (MJ/BW^0.75^)	124	0.63	0.15	0.33	1.08
ADG (g/kg BW^0.75^)	124	7.60	3.44	0.43	18.8
SRW (kg)	97	58.2	24.8	25.0	100
Z	97	0.38	0.11	0.19	0.67
Altitude (m)	121	417	563	19.0	2,355
**Arid region**					
ME intake (MJ/d)	93	9.61	4.64	0.58	18.4
ADG (g/d)	93	111	75.3	2.90	284
Average BW (kg)	93	28.0	10.4	9.21	51.3
MBW (kg^0.75^)	93	12.0	3.42	5.29	19.2
ME intake (MJ/BW^0.75^)	93	0.78	0.30	0.11	1.44
ADG (g/kg BW^0.75^)	93	8.73	4.99	0.35	23.5
SRW (kg)	88	54.0	18.5	25.0	100
Z	88	0.54	0.19	0.20	0.97
Altitude (m)	89	692	830	3	3,000
**Semi-arid region**					
ME intake (MJ/d)	176	8.61	3.49	2.50	26.4
ADG (g/d)	176	110	82.8	1.40	374
Average BW (kg)	176	23.2	6.60	13.4	55.6
MBW (kg^0.75^)	176	10.5	2.20	7.00	20.4
ME intake (MJ/BW^0.75^)	176	0.81	0.23	0.36	1.43
ADG (g/kg BW^0.75^)	176	9.94	6.47	0.11	31.9
SRW (kg)	154	49.0	18.2	26.0	113
Z	154	0.53	0.16	0.19	1.00
Altitude (m)	176	653	665	−230	2,319
**Mediterranean region**					
ME intake (MJ/d)	122	12.8	3.64	2.92	22.3
ADG (g/d)	122	198	61.6	64.0	330
Average BW (kg)	122	31.9	6.70	17.8	46.6
MBW (kg^0.75^)	122	13.4	2.12	8.67	17.8
ME intake (MJ/BW^0.75^)	122	0.95	0.18	0.22	1.40
ADG (g/kg BW^0.75^)	122	14.9	4.22	4.47	23.8
SRW (kg)	101	58.9	19.8	32.5	112.5
Z	101	0.58	0.15	0.25	0.95
Altitude (m)	111	511	470	−230	1,650

^1^Metabolizable energy (ME), average daily gain (ADG), body weight (BW), and degree of maturity (Z) given by the ratio of current weight /standard reference weight (SRW) at maturity (CSIRO, 2007).

^2^Number of observations.

### Species and Sex Effect on Metabolizable Energy Requirements

Results of the analysis of the effects of species and sex on estimates of ME requirements are presented in Table 2. The MEm was not affected by species (Model 1; *P* = 0.50), with an average requirement of 0.428 (± 0.039) and 0.465 (± 0.035) MJ/kg^0.75^, for sheep and goats, respectively. However, the MEg (MJ/g ADG) was affected by species (*P = *0.02), being 0.032 (± 0.002) and 0.026 (± 0.002) MJ/g ADG for sheep and goats, respectively. In addition, the maturity degree was greater (*P < *0.01) for sheep compared to goats (0.54 ± 0.01 vs. 0.44 ± 0.02; results not presented). Thus, to evaluate the effect of sex on MEm and MEg, we analyzed the data from sheep and goats separately. The MEm was not affected by sex in sheep (*P* ≥ 0.76) and goats (*P *≥ 0.32). The Meg was not affected by sex in sheep (P ≥ 0.41), but it differed (P ≤ 0.05) between intact males and females in goats. Intact males had a greater MEg (Model 3; *P* = 0.02) compared to females (0.030 ± 0.003 vs. 0.013 ± 0.006 MJ/g ADG). The MEg did not differ between castrated males (*P* ≥ 0.34; 0.021 ± 0.006 MJ/g ADG) and intact males or females. The degree of maturity tended to be significant (*P = *0.09) when included in the complete dataset (including goats and sheep), but it was not significant in the sheep dataset (*P *= 0.31) nor in the goat dataset (*P *= 0.34) when we included it along with the factor variable sex.

**Table 2.** Effects of species and sex on metabolizable energy requirements for maintenance (MEm; MJ/kg^0.75^ BW) and weight gain (MEg; MJ/g ADG) of sheep and goats

Item^1^	Specie effect	Sex effect^2^	Sex effect^3^	Contrasts^4^			
Model n.	1	2	3	G vs.S	M vs. F	M vs. C	F vs. C
** Intercept**							
Goat	0.465 ± 0.035 (<0.01)			0.50			
Sheep	0.428 ± 0.039 (<0.01)						
* Sheep specie*							
Intact male		0.432 ± 0.048 (<0.01)			0.85	0.88	0.76
Female		0.538 ± 0.129 (<0.01)					
Castrated male		0.479 ± 0.110 (<0.01)					
* Goat specie*							
Intact male			0.528 ± 0.059 (<0.01)		0.32	0.95	0.34
Female			0.751 ± 0.087 (<0.01)				
Castrated male			0.578 ± 0.083 (<0.01)				
** Slope**							
Goat	0.026 ± 0.002 (<0.01)			0.02			
Sheep	0.032 ± 0.002 (<0.01)						
* Sheep specie*							
Intact male		0.032 ± 0.002 (<0.01)			0.76	0.41	0.99
Female		0.024 ± 0.011 (0.03)					
Castrated male		0.024 ± 0.006 (<0.01)					
* Goat specie*							
Intact male			0.030 ± 0.003 (<0.01)		0.02	0.34	0.64
Female			0.013 ± 0.006 (0.02)				
Castrated male			0.021 ± 0.006 (<0.01)				
Z	0.105 ± 0.06 (0.09)	0.086 ± 0.085 (0.31)	−0.110 ± 0.115 (0.34)				
* n*	850	491	233				

^1^Z is the degree of maturity given by the ratio of current weight /standard reference weight at maturity (CSIRO, 2007).

^2^Model 2 includes sheep only.

^3^Model 3 includes goats only.

^4^Contrasts to compare the intercepts and slopes of models predicting metabolizable energy requirements in males (M), females (F), and castrated (C) sheep (S) and goats (G) using the Tukey test from the emmeans R package. The intercept represents the MEm (MJ/kg^0.75^ BW), and the slope represents the MEg (MJ/g ADG).

### Metabolizable Energy Requirements by Climatic Region

The triple interaction between climatic region × specie × ADG was not significant (*P = *0.33). Thus, we decided to pool the data sets of sheep and goats for analyzing the impacts of climate on MEm and MEg. Metabolizable energy requirements for maintenance and MEg were both affected (*P* < 0.05) by the climatic region in which the animals were raised (Tables 4 and 5). Small ruminants raised in subtropical regions had a lower (*P *= 0.03; Model 4) MEm (0.497 ± 0.046 MJ/kg^0.75^) than those in semi-arid (0.600 ± 0.038 MJ/kg^0.75^) and tended (*P *= 0.08) to have a lower MEm than those raised in arid regions (0.529 ± 0.050 MJ/kg^0.75^). However, these differences disappeared when the intercepts were adjusted for the differences in the degree of maturity (model 5). In addition, when adjusted for diet composition and digestibility (model 6) or altitude (model 7), the differences between MEm between small ruminants raised in subtropical and arid regions disappeared. However, the differences between those raised in semiarid and subtropical regions remained even after adjusting for diet composition and digestibility (model 6) or altitude (model 7). Small ruminants raised in Mediterranean regions tended (*P = *0.07) to have greater MEm than those raised in subtropical conditions.

**Table 4.** Contrasts of intercepts and slopes of models predicting energy requirements of sheep and goats raised in different climatic regions from Table 3

Models	CE^2^	CE + Z^3^	CE + DCD^4^	CE + Altitude
	4	5	6	7
Contrasts^1^				
**Intercepts**				
A vs. M	0.99	0.97	0.99	0.99
A vs. SA	0.99	0.99	0.99	0.99
A vs. ST	0.08	0.60	0.29	0.11
A vs. T	0.35	0.79	0.92	0.45
M vs. SA	0.99	0.99	0.99	0.99
M vs. ST	0.22	0.25	0.07	0.32
M vs. T	0.68	0.34	0.62	0.84
SA vs. ST	0.03	0.28	0.05	0.04
SA vs. T	0.15	0.33	0.54	0.19
ST vs. T	0.65	0.95	0.30	0.64
**Slopes**				
A vs. M	0.91	0.16	0.02	0.98
A vs. SA	0.52	0.39	0.04	0.64
A vs. ST	0.56	0.61	0.11	0.65
A vs. T	0.99	0.99	0.75	0.98
M vs. SA	0.95	0.94	0.96	0.90
M vs. ST	0.92	0.99	0.99	0.86
M vs. T	0.57	0.01	0.02	0.61
SA vs. ST	0.99	0.99	0.99	0.99
SA vs. T	0.03	0.03	0.02	0.02
ST vs. T	0.23	0.35	0.22	0.19

^1^Contrasts to compare the intercepts and slopes of models predicting metabolizable energy requirements in sheep and goats raised in arid (A), mediterranean (M), semi-arid (SA), subtropical, (ST), tropical (T) conditions using the Tukey test from the emmeans R package.

^2^CE: climate effect.

^3^Z: degree of maturity given by the ratio of current weight /standard reference weight at maturity (CSIRO, 2007).

^4^Diet composition and digestibility.

**Table 5.** Prediction equations of metabolizable energy requirements for maintenance (MEm; MJ/kg^0.75^ BW) and weight gain (MEg; MJ/g ADG) of sheep and goats raised in various climatic regions

Item	Global	Tropical	Subtropical	Arid	Semi-arid	Mediterranean
Model n.	8	9	10	11	12	13
** Intercept**	0.487 ± 0.016 (<0.01)	0.446 ± 0.021 (<0.01)	0.488 ± 0.033 (<0.01)	0.539 ± 0.07 (<0.01)	0.599 ± 0.042 (<0.01)	0.569 ± 0.057 (<0.01)
** Slope**	0.030 ± 0.001 (<0.01)	0.032 ± 0.002 (<0.01)	0.022 ± 0.003 (<0.01)	0.030 ± 0.004 (<0.01)	0.023 ± 0.003 (<0.01)	0.025 ± 0.004 (<0.01)
* n*	992	477	124	93	176	122
*Monte Carlo cross-evaluation* ^1^						
RMSE	0.19	0.18	0.17	0.26	0.20	0.16
RMSPE	25.6	25.5	26.6	34.7	25.4	16.7
RSR	0.79	0.80	1.14	0.88	0.91	0.89
* Mean bias*	2.13	3.63	10.7	14.0	8.87	8.49
* Slope bias*	1.35	2.95	14.2	7.16	5.96	8.52
CCC	0.57	0.58	0.11	0.42	0.44	0.44

^1^Root mean square error (RMSE), root mean square percentage error (RMSPE), RMSPE-observations standard deviation ratio (RSR), and concordance correlation coefficient (CCC). The intercept represents the MEm (MJ/kg^0.75^ BW), and the slope represents the MEg (MJ/g ADG).

The MEg for small ruminants raised in arid regions was significantly (*P* < 0.05) greater (0.032 ± 0.006 MJ/g ADG) than those raised in the Mediterranean (0.009 ± 0.004 MJ/g ADG) or semi-arid regions (0.009 ± 0.004 MJ/g ADG), when the slopes were adjusted for the diet composition and digestibility (Model 6). In addition, small ruminants raised in Mediterranean regions also had lower (*P < *0.05) MEg (0.019 ± 0.004 and 0.009 ± 0.004 MJ/g ADG in models 5 and 6, respectively) than those raised in tropical regions (0.033 ± 0.002 and 0.024 ± 0.002 MJ/g ADG in models 5 and 6, respectively) when the respective slopes were adjusted for variations in the maturity of degree, diet composition and digestibility across regions. The MEg of animals raised in semi-arid regions was consistently lower than those raised in tropical conditions independent of the co-variables tested in the models derived in this meta-analysis (Tables 3 and 4). Altitude (*P = *0.72) and Z (*P = *0.32) were not significant when offered in regression models looking at the effect of climate region on MEm and MEg. However, given the biological relevance of Z on energy requirements (CSIRO, 2007), and since it was statistically different between climatic regions (Table S2), we decided to evaluate the impact of this variable in the model predicting energy requirements in different climate regions (Model 5; Table 3).

**Table 3.** Effects of climatic region on metabolizable energy requirements for maintenance (MEm; MJ/kg^0.75^ BW) and weight gain (MEg; MJ/g ADG) of sheep and goats

Item^1^	CE^2^	CE + Z	CE + DCD^3^	CE + Altitude
Model n.	4	5	6	7
** Intercept**				
Tropical	0.445 ± 0.022 (<0.01)	0.412 ± 0.036 (<0.01)	0.156 ± 0.080 (0.05)	0.444 ± 0.026 (<0.01)
Subtropical	0.497 ± 0.046 (<0.01)	0.481 ± 0.057 (<0.01)	0.173 ± 0.098 (0.08)	0.501 ± 0.048 (<0.01)
Arid	0.529 ± 0.05 (<0.01)	0.467 ± 0.063 (<0.01)	0.141 ± 0.118 (0.24)	0.538 ± 0.056 (<0.01)
Semi-arid	0.600 ± 0.038 (<0.01)	0.568 ± 0.053 (<0.01)	0.326 ± 0.084 (<0.01)	0.601 ± 0.039 (<0.01)
Mediterranean	0.553 ± 0.062 (<0.01)	0.623 ± 0.074 (<0.01)	0.369 ± 0.108 (<0.01)	0.540 ± 0.064 (<0.01)
** Slope**				
Tropical	0.032 ± 0.002 (<0.01)	0.033 ± 0.002 (<0.01)	0.024 ± 0.002 (<0.01)	0.033 ± 0.002 (<0.01)
Subtropical	0.021 ± 0.005 (<0.01)	0.022 ± 0.006 (<0.01)	0.012 ± 0.006 (0.04)	0.021 ± 0.005 (<0.01)
Arid	0.031 ± 0.004 (< 0.01)	0.033 ± 0.004 (<0.01)	0.032 ± 0.006 (<0.01)	0.030 ± 0.004 (<0.01)
Semi-arid	0.023 ± 0.003 (<0.01)	0.023 ± 0.003 (<0.01)	0.013 ± 0.003 (<0.01)	0.023 ± 0.003 (<0.01)
Mediterranean	0.026 ± 0.004 (<0.01)	0.019 ± 0.004 (<0.01)	0.009 ± 0.004 (0.04)	0.027 ± 0.004 (<0.01)
Z		0.062 ± 0.062 (0.32)		
CP, % DM			−0.004 ± 0.001 (<0.01)	
NDF, % DM			−0.002 ± 0.001 (0.01)	
OMD, % DM			0.008 ± 0.001 (<0.01)	
Altitude, m				−0.000006 ± 0.00002 (0.72)
* n*	992	850	472	961

^1^Z: degree of maturity given by the ratio of current weight /standard reference weight at maturity (CSIRO, 2007); CP: crude protein content; NDF: neutral detergent fiber; OMD: organic matter digestibility; *n*: number of treatment means.

^2^CE: climate effect.

^3^Diet composition and digestibility. The intercept represents the MEm (MJ/kg^0.75^ BW), and the slope represents the MEg (MJ/g ADG).

### Models Predicting Metabolizable Energy Requirements for Maintenance and Gain

**Figure 2.**
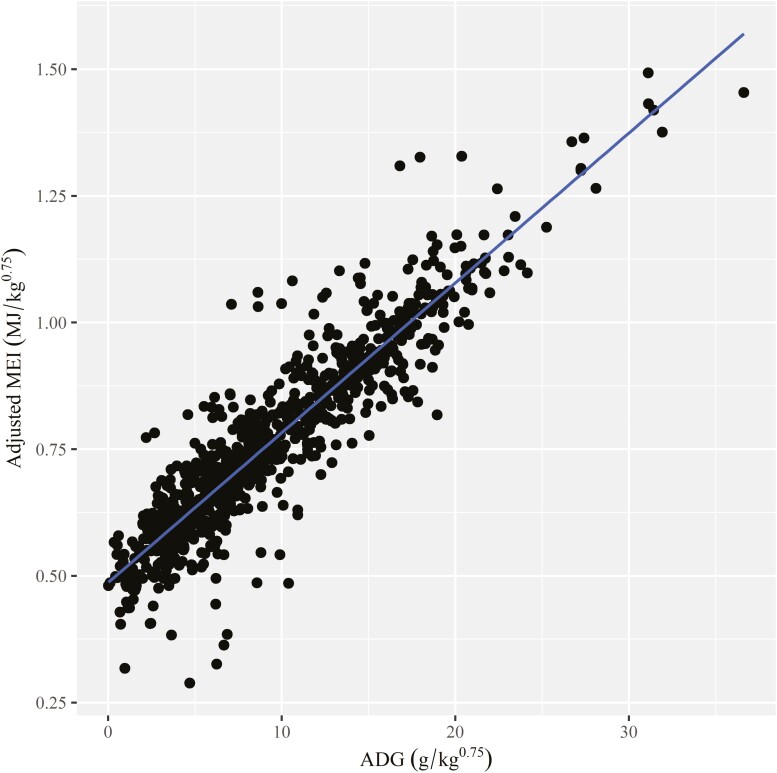
Relationship between metabolizable energy intake (MEI) and average daily gain (ADG) in the Global model (n = 992). Metabolizable energy intake observations were adjusted for the random study effect (St-Pierre, 2001).

**Figure 3.**
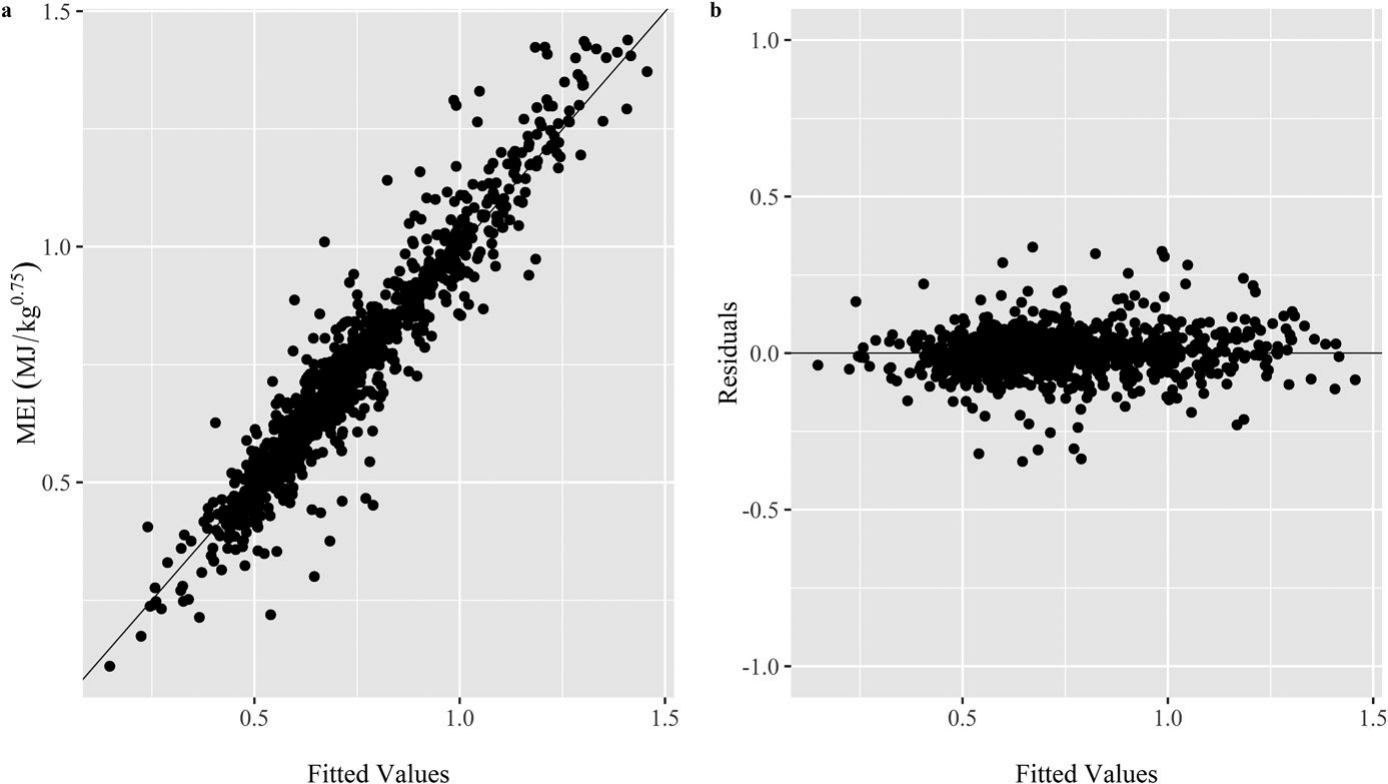
Observed versus predicted (panel a) and residuals versus predicted (panel b) values for metabolizable energy intake (MJ/kg^0.75^) in the Global model. Each point represents a treatment mean (n = 992).

Models predicting MEm and MEg for small ruminants raised in various climatic regions, along with the results of Monte Carlo cross-evaluation, are given in Table 5. The global model, which included our entire database (Model 8), had the lowest RSR, mean bias, and slope bias to the models derived specifically for climate regions. Plots of the relationship between adjusted MEI (MJ/kg^0.75^) and ADG (g/kg^0.75^) and of observed, predicted, and residual values for the global model (Model 8) are presented in Figures 2 and 3, respectively. The equation generated for the tropical region (Model 9 and Figures S4 and S5) also had similar performance to the Global equation and showed a considerably lower MEm than reported for the Global equation (Model 8). In addition, there were no benefits in deriving models specific for each sex because of similar performance exhibited by Model S1 for intact males and worse performance by Models S2 and S3 for females and castrated males in sheep, respectively (Table S3). In addition, models derived by sex in goats had considerable mean and slope bias (Table S4). Because of the low number of observations for sex within species (e.g., female sheep), the interactions between sex and climate regions were not assessed.

## DISCUSSION

### Overview and Limitations

In this meta-analysis, models were derived to predict MEm and MEg in small ruminants of different sexes and raised in contrasting climatic regions. We also derived models to evaluate the effects of species, sex, and climatic region on metabolizable energy requirements in small ruminants. Net energy is the actual energy remaining after fermentation, digestion, absorption and metabolism and is the form of energy available for maintenance and production (NRC, 2007). However, NE is difficult to measure because it requires feeding the animal and collecting all excreta, gas and heat production for each feed available. As a consequence, very few studies in our dataset reported NE values. Therefore, the method dose-response was used to estimate ME requirements based on a meta-analysis of feeding trials. Indeed, dose-response studies have been broadly used to estimate nutritional requirements (NRC, 2007). We estimated the MEm and MEg using linear mixed-effects regression models of MEI (MJ/kg^0.75^) as a function of ADG (g/kg^0.75^) as proposed by Luo et al. (2004), which might lead to different estimates compared to more traditional methods such as comparative slaughter techniques and calorimetry (Souza et al., 2020; Moon et al., 2021). In addition, ME values reported in the original publications may be unreliable. In most cases, authors relied on tabular values to estimate ME values for the diets, and there was no way for us to evaluate the criteria adopted for each author in their respective studies. The majority of studies in our database were feeding trials that did not measure body composition nor the mature weight of the breed used, we had to rely on reference values for the mature weight of the breeds included in our database. Finally, the low number of observations observed for some climate regions, such as in the arid regions, might have prevented us from finding significant differences in MEm compared to the other climate regions. However, we employed rigorous and standardized statistical methodologies tailored specifically for meta-analysis, ensuring the robustness of our findings and providing a broad interpretation of the impact of climatic regions on ME energy requirements in small ruminants. In addition, this specific type of evaluation, considering climatic differences around the world, would be very difficult to conduct in an individual study. Given the inherent limitations regarding the absence of large-scale experiments assessing body composition, mature weight across various breeds, and the influence of climatic variables alongside measurements of ME or NE, there may be little room to improve upon the current models. Nonetheless, our models offer valuable insights into potential shifts in maintenance energy requirements across different climatic regions.

### Species and Sex Effects on Metabolizable Energy Requirements

There are no extensive comparisons of energy requirements between sheep and goats and results are inconsistent. For instance, the AFRC (1998) suggested that the MEm of goats, scaled by body weight (BW)^0.75^, is greater than that of sheep. However, Salah et al. (2014) found no differences in MEm and MEg between sheep and goats. The greater MEg observed for sheep in our database can be explained by the greater maturity degree observed for sheep compared to goats in this meta-analysis (NRC, 2007; BR-Caprinos & Ovinos, 2024). Differences in body composition between sexes may lead to different energy requirements in growing small ruminants (Almeida et al., 2016). In growing goats at similar BW, females have greater body fat and lower body protein compared to intact and castrated males because they reach maturity at an early stage (Almeida et al., 2016). Thus, a lower energy requirement for maintenance is expected for females because the efficiency of protein deposition is lower than fat deposition, which implies lower maintenance costs (Garrett, 1980). Souza et al. (2020) evaluated energy requirements in growing goats from different sexes using individual data from 7 comparative slaughter studies and observed greater NEm and MEm in intact and castrated males compared to females using the comparative slaughter technique. However, the authors were unable to detect differences between sexes when they evaluated NEm and MEm through feeding trials, which was the approach adopted in our meta-analysis. Also, when Souza et al. (2020) considered body composition in their models through the ratio of empty BW to mature empty BW, the differences between sexes were no longer significant. Similarly, in this meta-analysis, no differences in MEm were found between intact male, female, and castrated males in goats or sheep, which is in line with the results reported in the literature for goats (NRC, 2007; Bompadre et al., 2014; Souza et al., 2020). Body composition significantly influences energy requirements for growth (NRC, 2007). It is well documented in small ruminants that females start fat deposition at an early stage compared to intact or castrated males (BR-Caprinos & Ovinos, 2024). Therefore, gain composition is crucial in understanding sex-based energy requirement variations. As expected, when the degree of maturity was considered in model 2, no differences for MEg were found in sheep as the differences in body composition were accounted for through the maturity degree (Souza et al., 2017). However, we found greater energy requirements for intact males compared to females in goats, which contradicts the biological expectation. It is important to highlight that given the fact that the majority of studies in our database were feeding trials that did not measure body composition nor the mature weight of the breed used, we had to rely on reference values for the mature weight of the breeds included in our database. Mature weight in small ruminants is influenced by genetic factors, breed characteristics, and environmental conditions, including nutrition and management practices (Mahgoub et al., 2012). These factors interact to determine the growth potential and final mature size of small ruminants. Therefore, using an estimated mature weight without considering the factors described above may have led to an inaccurate estimate of the maturity degree, which may not have properly corrected the differences in body composition between sexes in the goat database. Therefore, it is essential to further investigate body composition in order to accurately describe the mature body weight of goats from various genotypes before integrating it into energy requirement models (BR-Caprinos & Ovinos, 2024; Teixeira et al., 2024).

### Metabolizable Energy Requirements of Animals Raised in Regions With Contrasting Climatic Regions

To our knowledge, this is the first meta-analysis to examine differences in MEm and MEg due to the variation in climatic regions in small ruminants. Since there was no difference between diet composition, digestibility, and mature weight between small ruminants raised in subtropical, arid, and semi-arid regions, the lower MEm observed for small ruminants raised in subtropical regions could be related to environmental factors typical of subtropical climates. For instance, higher temperatures, typical of arid and semi-arid regions, may contribute to a reduced ability to dissipate heat, increasing the overall maintenance energy requirements due to thermal stress (NRC, 2007). However, although diet composition and digestibility were not significantly different between these climatic regions (Table S2), the differences in MEm disappeared when the intercepts were adjusted by these factors, which are known to affect MEm (NRC, 2007). According to (NRC, 2007), the level of feed intake is well recognized as one of the main effectors affecting MEm. Small ruminants raised in semi-arid, arid, and Mediterranean had greater ME intake levels than those raised in subtropical regions (Table S2). Also, the fact that small ruminants raised in arid, semi-arid, and Mediterranean regions had greater maturity degrees than those raised in subtropical conditions (Table S2), which presumably would lead to a lower MEm (Cannas et al., 2006), was not observed in our study. Taken together, these findings suggest that differences in environmental and dietary factors may have had a greater impact than the differences in the maturity degree between small ruminants raised in subtropical and arid, semi-arid, and Mediterranean climatic regions.

The absence of significant differences in MEm for animals raised in arid with other climatic regions was unexpected and is likely associated with the low number of observations included for this climatic region (n = 93) in our dataset compared with tropical climate (n = 477). Even though our results indicate that small ruminants raised in Mediterranean regions had a higher degree of maturity (Table S2), the greater MEg for animals raised in tropical regions could be attributed to a greater fat deposition in genotypes raised in the tropical areas (Salah et al., 2014). Our estimates of MEg for small ruminants raised in tropical (0.033 ± 0.002 MJ/g ADG) and arid (0.031 ± 0.004 MJ/g ADG) conditions were close to those reported by Early et al. (2001) for ram lambs in hot climates (0.034 ± 0.005). There is a pressing need for energy requirement models specifically tailored to hot climates, as emphasized by Teixeira et al. (2024), particularly in light of climate change and the increasing frequency and intensity of heat waves.

### Prediction Equations of Metabolizable Energy Requirements

Because of the significant effects of species, sex, and climate on MEm and/or MEg, subsets of data for each sex and climate condition were created to evaluate whether the prediction accuracy of the models would increase by deriving specific models for each sex and climatic region. Interaction between species, climatic region, and sex was not assessed in our data because of the incomplete factorial overlap of species, climatic region, and sex in our dataset. Results indicate no benefits of developing models specific for climatic regions or sex (Tables S3 and S4) to predict ME requirements compared to a global model. The lack of benefits of deriving models that are climatic region-specific to predict energy requirements could be explained by the low number of observations for some regions, such as the subtropical and arid climates, which led to greater mean and slope biases. The equation generated for the tropical region also had similar performance to the Global equation and showed a considerably lower MEm than reported for the Global equation (Model 8), and for current feeding systems, which reported 0.536 MJ/kg^0.75^ (NRC, 2007) and 0.490 MJ/kg^0.75^ (AFRC, 1998) for goats in North America and United Kingdom, respectively. In addition, our estimate for MEm in tropical regions was very close to that recommended by the recent BR-Caprinos & Ovinos (2024), which reported 0.440 MJ/kg^0.75^ for an intact male goat weighing 30 kg gaining 200 g/d. The Brazilian system was developed using data from goats raised under tropical conditions and employing the comparative slaughter technique (BR-Caprinos & Ovinos, 2024). Our estimates of MEg in the Global and tropical models were slightly higher than those recommended by the NRC (2007) and the BR-Caprinos & Ovinos (2024) for growing goats, which reported 0.023 and 0.022 MJ/g ADG, respectively. These differences may be related to the methods used to estimate energy requirements as well as differences in the body composition of the animals used to derive requirement equations by each nutritional system. Given the broader range of diets, breeds, and environmental conditions included in our database, the global and tropical models developed in this study are recommended for use in ration formulation software for small ruminants raised in the tropical and subtropical zones, with the latter targeted towards small ruminants in tropical regions.

## CONCLUSIONS

This meta-analysis provides a comprehensive evaluation of species-specific differences in ME requirements in small ruminants while recognizing the challenges posed by confounding effects and climatic variability inherent in global datasets. The results of the current meta-analysis indicate considerable differences in the MEm and MEg in goats and sheep raised under different climatic regions. Notably, animals raised under subtropical conditions had a lower MEm than those in semi-arid and Mediterranean regions. In addition, the MEg for animals raised under Mediterranean conditions was lower than those raised in arid or tropical conditions. Using models predicting energy requirements taking into account climatic regions should lead to improved accuracy in feeding diets to small ruminants that more closely meet their energy requirements. However, this could not be confirmed in our study. Our analysis indicates that animals raised in tropical conditions may have lower MEm than recommended by current feeding systems, especially for those developed primarily using data obtained in temperate climates. Further research is needed to determine the mature size of different breeds raised in different climate conditions and how nutritional and environmental factors in those regions affect the mature size. In addition, energy requirement models tailored for hot climates are needed.

## Supplementary Material

txaf012_suppl_Supplementary_Material
