# Supplementary material for: Energy requirements of growing small ruminants raised for meat production in contrasting climatic regions: a meta-analysis
Source: Transl Anim Sci. 2025 Feb 5;9:txaf012. doi: 10.1093/tas/txaf012 (PMC11879033; doi:10.1093/tas/txaf012)
Supplement: txaf012_suppl_Supplementary_Material [file txaf012_suppl_supplementary_material.docx]

**Energy requirements of growing small ruminants raised for meat production in contrasting climatic regions: a meta-analysis**

V. C. Souza†^,*^, A. Bougouin†, H. Archimede^§^, A. Adegbola^‡^ and E. Kebreab†

†Department of Animal Science, University of California, Davis, CA 95616, USA

^§^INRAE, Unité de Recherches Zootechniques, 97170, Petit-Bourg, France

^‡^Department of Animal Sciences, University of Florida, Gainesville, FL 32611, USA

*Corresponding author: [vinicius2042@hotmail.com](mailto:vinicius2042@hotmail.com)

**Supplemental materials**

**Table S1.** Studies from the sheep dataset used for model development and evaluation.

| **Publication ID^1^** | **Reference** |
| --- | --- |
| 3 | Haddad, S.G. 2006. Bitter vetch grains as a substitute for soybean meal for growing lambs. Livest. Sci., v.99, p.221-225. Doi: 10.1016/j.livprodsci.2005.06.014 |
| 4 | Dhakad, A., A.K. Garg, P. Singh, D.K. Agrawal. 2002. Effect of replacement of maize grain with wheat bran on the performance of growing lambs. Small Rumin. Res. v.43, p.227-234. Doi: 10.1016/S0921-4488(02)00025-1 |
| 5 | Mahgoub, O., C.D. Lu, R.J. Early. 2000. Effects of dietary energy density on feed intake, body weight gain and carcass chemical composition of Omani growing lambs. Small Rumin. Res. v.37, p.35-42. Doi: 10.1016/S0921-4488(99)00132-7 |
| 6 | Sheridan, R., A.V. Ferreira, L.C. Hoffman. 2003. Production efficiency of South African Mutton Merino lambs and Boer goat kids receiving either a low or a high energy feedlot diet. Small Rumin. Res. v.50, p.75-82. Doi: 10.1016/S0921-4488(03)00109-3 |
| 8 | Fimbres H., G. Hernández-Vidal, J.F. Picón-Rubio, J.R. Kawas, C.D. Lu. 2002. Productive performance and carcass characteristics of lambs fed finishing ration containing various forage levels. Small Rumin. Res. v.43, p.283-288. Doi: 10.1016/S0921-4488(02)00014-7 |
| 12 | Rokomatu, I., E.M. Aregheore. 2006. Effects of supplementation on voluntary dry matter intake, growth and nutrient digestibility of the Fiji Fantastic sheep on a basal diet of Guinea grass (Panicum maximum). Livest. Sci. v.100, p.132-141. Doi: 10.1016/j.livprodsci.2005.08.014 |
| 14 | Hue, K.T., Do T.T. Van, I. Ledin. 2008. Effect of supplementing urea treated rice straw and molasses with different forage species on the performance of lambs. Small Rumin. Res. v.78, p.134-143. Doi: 10.1016/j.smallrumres.2008.05.010 |
| 17 | Ngwa, A.T., I.V. Nsahlai, P.A. Iji. 2002. Effect of supplementing veld hay with a dry meal or silage from pods of Acacia sieberiana with or without wheat bran on voluntary intake, digestibility, excretion of purine derivatives, nitrogen utilization, and weight gain in South African Merino sheep. Livest. Prod. Sci. v.77, p.253-264. Doi: 10.1016/S0301-6226(02)00035-0 |
| 18 | Umunna, N.N., P.O. Osuji, I.V. Nsahlai, H. Khalili, M.A. Mohamed-Saleem. 1995. Effect of supplementing oat hay with lablab, sesbania, tagasaste or wheat middlings on voluntary intake, N utilization and weight gain of Ethiopian Menz sheep. Small Rumin. Res. v.18, p.113-120. Doi: 10.1016/0921-4488(95)00686-F |
| 19 | Moscoso, C., M. Vélez, A. Flores, N. Agudelo. 1995. Effects of guanacaste tree (Enterolobium cyclocarpum Jacq. Griseb.) fruit as replacement for sorghum grain and cotton-seed meal in lamb diets. Small Rumin. Res. v.18, p.121-124. Doi: 10.1016/0921-4488(95)00677-D |
| 21 | Olfaz, M., N. Ocak, G. Erener, M.A. Cam, A.V. Garipoglu. Growth, carcass and meat characteristics of Karayaka growing rams fed sugar beet pulp, partially substituting for grass hay as forage. Meat Sci. v.70, p.7-14. Doi: 10.1016/j.meatsci.2004.11.015 |
| 24 | Obeidat, B.S., A.Y. Abdullah, F.A. Al-Lataifeh. 2008. The effect of partial replacement of barley grains by Prosopis juliflora pods on growth performance, nutrient intake, digestibility, and carcass characteristics of Awassi lambs fed finishing diets. Anim. Feed Sci. Technol. v.146, p.42-54. Doi: 10.1016/j.anifeedsci.2007.12.002 |
| 25 | Awawdeh, M.S., B.S. Obeidat, A.Y. Abdullah, W.M. Hananeh. 2009. Effects of yellow grease or soybean oil on performance, nutrient digestibility and carcass characteristics of finishing Awassi lambs. Anim. Feed Sci. Technol. v.153, p.216-227. Doi: 10.1016/j.anifeedsci.2009.06.013 |
| 32 | Rocha, M.H.M.; I. Susin; A.V. Pires; J.S. Fernandes Jr.; C.Q. Mendes. 2004. Performance of Santa Ines lambs fed diets of variable crude protein levels. Sci. agric. v.61, p.141-145. Doi: 10.1590/S0103-90162004000200003 |
| 33 | Keser, O., T. Bilal, H. Can Kutay. 2008. The effect of different dietary crude protein level on performance and serum immunoglobulin G in male Kivircik lambs. Bulg. J. Vet. Med. v.11, p.49−54. |
| 35 | Yusuf, A.M., O.A. Olafadehan, C.O. Obun, M. Inuwa, M.H. Garba, S.M. Shagwa. Nutritional Evaluation of Sheabutter Fat in Fattening of Yankasa Sheep. Pak. J. Nutr. v.8, p.1062-1067. Doi: 10.3923/pjn.2009.1062.1067 |
| 37 | Matizha, W., N.T. Ngongoni, J.H. Topps. 1997. Effect of supplementing veld hay with tropical legumes Desmodium uncinatum, Stylosanthes guianensis and Macroptilium atropurpureum on intake, digestibility, outflow rates, nitrogen retention and live weight gain in lambs. Anim. Feed Sci. Technol. v.69, p.187-193. Doi: 10.1016/S0377-8401(97)81633-1 |
| 38 | Dey. A., N. Dutta, K. Sharma, A.K. Pattanaik. 2007. Effect of dietary supplementation of leaves as source of condensed tannins on the performance of lambs. Livest. Res. Rural. Dev. v.19. |
| 39 | Adebowale, E.A. 1981. The maize replacement value of fermented cassava peels (Manihot utilissma Pohl) in rations for sheep. Trop. Anim. Prod. v.6, p.54-59. |
| 40 | Tripathi, M.K., S.A. Karim, O.H. Chaturvedi, V.K. Singh. 2006. Effect of ad libitum tree leaves feeding with varying levels of concentrate on intake, microbial protein yield and growth of lambs. Livest. Res. Rural. Dev. v.18. |
| 41 | Madhavi, K., T.J. Reddy, Y. Ramana Reddy, G.V.N. Reddy. 2006. Effect of feeding differently processed detoxified neem (Azadirachta indica) seed cake based complete diet on growth, nutrient utilization and carcass characteristics in Nellore sheep. Livest. Res. Rural. Dev. v.18. |
| 43 | Nantoumé, H., C.H.T. Diarra, D. Traoré. 2006. Performance et rentabilité économique de l'incorporation des quatre fourrages de qualité pauvre dans des rations d'engraissement des moutons Maures. Livest. Res. Rural. Dev. v.18. |
| 45 | Olorunnisomo, O.A., M.K. Adewumi, O.J. Babayemi. 2006. Effects of nitrogen level on the utilization of maize offal and sorghum brewer's grain in sheep diets. Livest. Res. Rural. Dev. v.18. |
| 47 | Murro, J.K., V.R.M. Muhikambele, S.V. Sarwatt. 2003. Moringa oleifera leaf meal can replace cottonseed cake in the concentrate mix fed with Rhodes grass (Chloris gayana) hay for growing sheep. Livest. Res. Rural Dev. v.15 |
| 50 | Patel, K.M., K.S. Patel, K.N. Wadhwani, S. Parnerkar, P.R. Pandya, A.M. Patel. 2004. Comparative growth performance of weaner lambs on nonconventional based ration under intensive production system. Int. J. Agri. Biol. v.6, p.860-864. |
| 52 | Abdullah, A.Y., M.M. Muwalla, R.I. Qudsieh, H.H. Titi. 2010. Effect of bitter vetch (Vicia ervilia) seeds as a replacement protein source of soybean meal on performance and carcass characteristics of finishing Awassi lambs. Trop. Anim. Health Prod. v.42, p.293-300. Doi: 10.1007/s11250-009-9420-x |
| 53 | García-Winder, L.R., S. Goñi-Cedeño, P.A. Olguín-Lara, G. Díaz-Salgado, C.M. Arriaga-Jordán. 2009. Huizache (Acacia farnesiana) whole pods (flesh and seeds) as an alternative feed for sheep in Mexico. Trop. Anim. Health Prod. v.41, p.1615–1621. Doi: 10.1007/s11250-009-9355-2 |
| 54 | Al-Owaimer, A.N., S.M. Zahran, B.A. Al-Bassam. 2008. Effect of feeding some types of Atriplex ssp, in complete diet on growth performance and digestibility of growing lambs. Agric. Res. Bulletin No King Saud Univ Riyadh, Saudi Arabia. 161:5–19 |
| 58 | Omer, H.A.A., S.S. Abdel-Magid, S.M. Ahmed, M.I. Mohamed, I.M. Awadalla. 2010. Response to partial replacement of yellow corn with potato processing waste as non-traditional source of energy on the productive performance of Ossimi lambs. Trop. Anim. Health Prod. v.42, p.1195–1202. Doi: 10.1007/s11250-010-9548-8 |
| 59 | Nurfeta, A. 2009. Feed intake, digestibility, nitrogen utilization, and body weight change of sheep consuming wheat straw supplemented with local agricultural and agro-industrial by-products. Trop. Anim. Health Prod. v.42, p.815–824. Doi: 10.1007/s11250-009-9491-8 |
| 60 | Dessie, J., S. Melaku, F. Tegegne, K.J. Peters. 2010. Effect of supplementation of Simada sheep with graded levels of concentrate meal on feed intake, digestibility and body-weight parameters. Trop. Anim. Health Prod. v.42, p.841–848. Doi: 10.1007/s11250-009-9496-3 |
| 61 | Sultan, J.I., A. Javaid, M. Aslam. 2010. Nutrient digestibility and feedlot performance of lambs fed diets varying protein and energy contents. Trop. Anim. Health Prod. v.42, p.941–946. Doi: 10.1007/s11250-009-9511-8 |
| 62 | Tafa, A., S. Melaku, K.J. Peters. 2010. Supplementation with linseed (Linum usitatissimum) cake and/or wheat bran on feed utilization and carcass characteristics of Arsi-Bale sheep. Trop. Anim. Health Prod. v.42, p.677–685. Doi: 10.1007/s11250-009-9475-8 |
| 63 | Macías-Cruz, U., J.A. Quintero-Elisea, L. Avendaño-Reyes, A. Correa-Calderón, F.D. Alvarez-Valenzuela, S.A. Soto-Navarro, F.A. Lucero-Magaña, A. González-Reyna. 2010. Buffel grass (Cenchrus ciliaria L.) substitution for orange pulp on intake, digestibility, and performance of hairsheep lambs. Trop. Anim. Health Prod. v.42, p.223–232. Doi: 10.1007/s11250-009-9410-z |
| 64 | Nega, A., Melaku, S. 2009. Feed intake, digestibility and body weight change in Farta sheep fed hay supplemented with rice bran and/or noug seed (Guizotia abyssinica) meal. Trop. Anim. Health Prod. v.41, p.507–515. Doi: 10.1007/s11250-008-9215-5 |
| 65 | Hagos, T., Melaku, S. 2009. Feed intake, digestibility, body weight and carcass parameters of Afar rams fed tef (Eragrostis tef) straw supplemented with graded levels of concentrate mix. Trop. Anim. Health Prod. v.41, p.599–606. Doi: 10.1007/s11250-008-9230-6 |
| 66 | Bishaw, F., S. Melaku, S. Effects of supplementation of Farta sheep fed hay with sole or mixtures of noug seed meal and wheat bran on feed intake, digestibility and body weight change. Trop. Anim. Health Prod. v.40, p.597–606. Doi: 10.1007/s11250-008-9138-1 |
| 67 | Baloyi, J.J., N.T. Ngongoni, H. Hamudikuwanda. 2008. The effect feeding forage legumes as nitrogen supplement on growth performance of sheep. Trop. Anim. Health Prod. v.40, p.457–462. Doi: 10.1007/s11250-007-9120-3 |
| 68 | Ayantunde, A.A., P. Delfosse, S. Fernandez-Rivera, B. Gerard, A. Dan-Gomma. Supplementation with groundnut haulms for sheep fattening in the West African Sahel. Trop. Anim. Health Prod. v.39, p.207–216. Doi: 10.1007/s11250-007-9009-1 |
| 69 | Abate, D., S. Melaku. 2009. Effect of supplementing urea-treated barley straw with lucerne or vetch hays on feed intake, digestibility and growth of Arsi Bale Sheep. Trop. Anim. Health Prod. v.41, p.579–586. Doi: 10.1007/s11250-008-9227-1 |
| 71 | Adjorlolo, L.K., K. Amaning-Kwarteng, F.K. Fianu. 2004. Preference of sheep for three forms of mucuna forage and the effect of supplementation with mucuna forage on the performance of sheep. Trop. Anim. Health Prod. v.36, p.145–156. Doi: 10.1023/b:trop.0000012102.42938.90 |
| 74 | Obeidat, B.S., B.H. Aloqaily. 2010. Using sesame hulls in Awassi lambs diets: Its effect on growth performance and carcass characteristics and meat quality. Small Rumin. Res. v.91, p.225–230. Doi: 10.1016/j.smallrumres.2010.03.004 |
| 75 | Ktita, S.R., A. Chermiti, M. Mahouachi. 2010. The use of seaweeds (Ruppia maritima and Chaetomorpha linum) for lamb fattening during drought periods. Small Rumin. Res. v.91, p.116–119. Doi: 10.1016/j.smallrumres.2010.01.012 |
| 76 | Obeidat, B.S., A.Y. Abdullah, K.Z. Mahmoud, M.S. Awawdeh, N.Z. Al-beitawi, F.A. Al-Lataifeh. 2009. Effects of feeding sesame meal on growth performance, nutrient digestibility, and carcass characteristics of Awassi lambs. Small Rumin. Res. v.82, p.13–17. Doi: 10.1016/j.smallrumres.2009.01.002 |
| 77 | Haddad, S.G., M.A. Ata. 2009. Growth performance of lambs fed on diets varying in concentrate and wheat straw. Small Rumin. Res. v.81, p.96–99. Doi: 10.1016/j.smallrumres.2008.11.015 |
| 78 | Dutta, T.K., M.K. Agnihotri, S.B.N. Rao. 2008. Effect of supplemental palm oil on nutrient utilization, feeding economics and carcass characteristics in post-weaned Muzafarnagari lambs under feedlot condition. Small Rumin. Res. v.78, p.66–73. Doi: 10.1016/j.smallrumres.2008.05.002 |
| 79 | Dey, A., N. Dutta, K. Sharma, A.K. Pattanaik. 2008. Effect of dietary inclusion of Ficus infectoria leaves as a protectant of proteins on the performance of lambs. Small Rumin. Res. v.75, p.105–114. Doi: 10.1016/j.smallrumres.2007.06.013 |
| 80 | Haddad, S.G., R.E. Nasr. 2007. Partial replacement of barley grain for corn grain: Associative effects on lambs’ growth performance. Small Rumin. Res. v.72, p.92–95. Doi: 10.1016/j.smallrumres.2006.08.005 |
| 81 | Tegegne, F., C. Kijora, K.J. Peters. 2007. Study on the optimal level of cactus pear (Opuntia ficus-indica) supplementation to sheep and its contribution as source of water. Small Rumin. Res. v.72, p.157–164. Doi: 10.1016/j.smallrumres.2006.10.004 |
| 82 | Tripathi, M.K., O.H. Chaturvedi, S.A. Karim, V.K. Singh, S.L. Sisodiya. 2007. Effect of different levels of concentrate allowances on rumen fluid pH, nutrient digestion, nitrogen retention and growth performance of weaner lambs. Small Rumin. Res. v.72, p.178–186. Doi: 10.1016/j.smallrumres.2006.10.008 |
| 84 | Galina, M.A., M. Guerrero, C.D. Puga. 2007. Fattening Pelibuey lambs with sugar cane tops and corn complemented with or without slow intake urea supplement. Small Rumin. Res. v.70, p.101–109. Doi: 10.1016/j.smallrumres.2006.01.014 |
| 86 | Bhatta, R., S. Vaithiyanathan, N.P. Singh, D.L. Verma. 2007. Effect of feeding complete diets containing graded levels of Prosopis cineraria leaves on feed intake, nutrient utilization and rumen fermentation in lambs and kids. Small Rumin. Res. v.67, p.75–83. Doi: 10.1016/j.smallrumres.2005.09.027 |
| 88 | Singh, S., S.S. Kundu, A.S. Negi, P.N. Singh. 2006. Cowpea (Vigna unguiculata) legume grains as protein source in the ration of growing sheep. Small Rumin. Res. v.64, p.247–254. Doi: 10.1016/j.smallrumres.2005.04.022 |
| 91 | Ben Salem, H., H. Abdouli, A. Nefzaoui, A. El-Mastouri, L. Ben Salem. 2005. Nutritive value, behaviour, and growth of Barbarine lambs fed on oldman saltbush (Atriplex nummularia L.) and supplemented or not with barley grains or spineless cactus (Opuntia ficus-indica f. inermis) pads. Small Rumin. Res. v.59, p.229–237. Doi: 10.1016/j.smallrumres.2005.05.010 |
| 92 | Haddad, S.G., K.Z. Mahmoud, H.A. Talfaha. 2005. Effect of varying levels of dietary undegradable protein on nutrient intake, digestibility and growth performance of Awassi lambs fed on high wheat straw diets. Small Rumin. Res. v.58, p.231–236. Doi: 10.1016/j.smallrumres.2004.10.005 |
| 94 | Al-Dabeeb, S.N. 2005. Effect of feeding low quality date palm on growth performance and apparent digestion coefficients in fattening Najdi sheep. Small Rumin. Res. v.57, p.37–42. Doi: 10.1016/j.smallrumres.2004.05.002 |
| 96 | Becholie, D., B. Tamir, T.H. Terrill, B.P. Singh, H. Kassa. 2005. Suitability of tagasaste (Chamaecytisus palmensis L.) as a source of protein supplement to a tropical grass hay fed to lambs. Small Rumin. Res. v.56, p.55–64. Doi: 10.1016/j.smallrumres.2004.02.012 |
| 97 | Galina, M.A., J.D. Hummel, M. Sánchez, G.F.W. Haenlein. 2004. Fattening Rambouillet lambs with corn stubble or alfalfa, slow intake urea supplementation or balanced concentrate. Small Rumin. Res. v.53, p.89–98. Doi: 10.1016/j.smallrumres.2003.08.008 |
| 98 | Irshaid, R.H., M.Y. Harb, H.H. Titi. 2003. Replacing soybean meal with sunflower seed meal in the ration of Awassi ewes and lambs. Small Rumin. Res. v.50, p.109–116. Doi: 10.1016/S0921-4488(03)00118-4 |
| 101 | Anil Kumar, G.K., V.S. Panwar, K.R. Yadav, S. Sihag. 2002. Mustard cake as a source of dietary protein for growing lambs. Small Rumin. Res. v.44, p.47–51. Doi: 10.1016/S0921-4488(02)00034-2 |
| 104 | Musalia, L.M., S. Anandan, V.R.B. Sastry, D.K. Agrawal. 2000. Urea-treated neem (Azadirachta indica A. juss) seed kernel cake as a protein supplement for lambs. Small Rumin. Res. v.35, p.107–116. Doi: 10.1016/S0921-4488(99)00085-1 |
| 105 | Al Jassim, R.A.M., K.I. Ereifej, R.A. Shibli, A. Abudabos. 1998. Utilization of concentrate diets containing acorns (Quercus aegilops and Quercus coccifera) and urea by growing Awassi lambs. Small Rumin. Res. v.29, p.289–293. Doi: 10.1016/S0921-4488(97)00124-7 |
| 109 | Khan, A.G., A. Azim, M.A. Nadeem, M.A. Khan. 1997. Effect of growing fattening diets on the growth performance of intensified Afghani lambs. Small Rumin. Res. v.25, p.39–42. Doi: 10.1016/S0921-4488(96)00963-7 |
| 110 | Chandrasekharaiah, M., M.R. Reddy, G.V.N. Reddy. 1996. Effect of feeding urea treated maize stover on growth and nutrient utilization by sheep and goats. Small Rumin. Res. v.22, p.141–147. Doi: 10.1016/S0921-4488(96)00864-4 |
| 111 | Al Jassim, R.A.M., S.A. Hassan, A.N. Al-Ani. 1996. Metabolizable energy requirements for maintenance and growth of Awassi lambs. Small Rumin. Res. v.20, p.239–245. Doi: 10.1016/0921-4488(95)00775-X |
| 114 | Ravikala, K., A.M. Patel, K.S. Murthy, K.N. Wadhwani. 1995. Growth efficiency in feedlot lambs on Prosopis juliflora based diets. Small Rumin. Res. v.16, p.227–231. Doi: 10.1016/0921-4488(95)00639-3 |
| 117 | Nkosi, B.D., R. Meeske, D. Palic, T. Langa, K.J. Leeuw, I.B. Groenewald. 2009. Effects of ensiling whole crop maize with bacterial inoculants on the fermentation, aerobic stability, and growth performance of lambs. Anim. Feed Sci. Technol. v.154, p.193–203. Doi: 10.1016/j.anifeedsci.2009.09.009 |
| 118 | Tamir, B., G. Asefa. 2009. Effects of different forms of Acacia saligna leaves inclusion on feed intake, digestibility and body weight gain in lambs fed grass hay basal diet. Anim. Feed Sci. Technol. v.153, p.39–47. Doi: 10.1016/j.anifeedsci.2009.05.010 |
| 119 | Ben Salem, H., H.P.S. Makkar. 2009. Defatted Moringa oleifera seed meal as a feed additive for sheep. Anim. Feed Sci. Technol. v.150, p.27–33. Doi: 10.1016/j.anifeedsci.2008.07.007 |
| 120 | Soren, N.M., V.R.B. Sastry. 2009. Replacement of soybean meal with processed karanj (Pongamia glabra) cake on the balances of karanjin and nutrients, as well as microbial protein synthesis in growing lamb. Anim. Feed Sci. Technol. v.149, p.16–29. Doi: 10.1016/j.anifeedsci.2008.04.011 |
| 123 | Titi, H.H., Dmour, R.O., Abdullah, A.Y. 2008. Growth performance and carcass characteristics of Awassi lambs and Shami goat kids fed yeast culture in their finishing diet. Anim. Feed Sci. Technol. v.142, p.33–43. Doi: 10.1016/j.anifeedsci.2007.06.034 |
| 124 | Mahgoub, O., I.T. Kadim, M.H. Al-Busaidi, K. Annamalai, N.M. Al-Saqri. 2007. Effects of feeding ensiled date palm fronds and a by-product concentrate on performance and meat quality of Omani sheep. Anim. Feed Sci. Technol. v.135, p.210–221. Doi: 10.1016/j.anifeedsci.2006.07.011 |
| 129 | Mahgoub, O., I.T. Kadim, S.M. Al-Jufaili, N.M. Al-Saqry, K. Annamalai, A. Ritchie. 2005. Evaluation of sun-dried sardines as a protein supplement for Omani sheep. Anim. Feed Sci. Technol. v.120, p.245–257. Doi: 10.1016/j.anifeedsci.2005.01.013 |
| 130 | Mahgoub, O., I.T. Kadim, E.H. Johnson, A. Srikandakumar, N.M. Al-Saqri, A.S. Al-Abri, A. Ritchie. 2005. The use of a concentrate containing Meskit (Prosopis juliflora) pods and date palm by-products to replace commercial concentrate in diets of Omani sheep. Anim. Feed Sci. Technol. v.120, p.33–41. Doi: 10.1016/j.anifeedsci.2005.01.011 |
| 131 | Haddad, S.G., S.N. Goussous. 2005. Effect of yeast culture supplementation on nutrient intake, digestibility and growth performance of Awassi lambs. Anim. Feed Sci. Technol. v.118, p.343–348. Doi: 10.1016/j.anifeedsci.2004.10.003 |
| 132 | Melaku, S., K.J. Peters, A. Tegegne. 2004. Supplementation of Menz ewes with dried leaves of Lablab purpureus or graded levels of Leucaena pallida 14203 and Sesbania sesban 1198: effects on feed intake, live weight gain and estrous cycle. Anim. Feed Sci. Technol. v.113, p.39–51. Doi: 10.1016/j.anifeedsci.2003.11.003 |
| 133 | Haddad, S.G., H.M. Younis. 2004. The effect of adding ruminally protected fat in fattening diets on nutrient intake, digestibility and growth performance of Awassi lambs. Anim. Feed Sci. Technol. v.113, p.61–69. Doi: 10.1016/j.anifeedsci.2003.10.015 |
| 134 | Tripathi, M.K., A. Santra, O.H. Chaturvedi, S.A. Karim. 2004. Effect of sodium bicarbonate supplementation on ruminal fluid pH, feed intake, nutrient utilization and growth of lambs fed high concentrate diets. Anim. Feed Sci. Technol. v.111, p.27–39. Doi: 10.1016/j.anifeedsci.2003.07.004 |
| 137 | Ben Salem, H., A. Nefzaoui, L. Ben Salem. 2002. Supplementation of Acacia cyanophylla Lindl. foliage-based diets with barley or shrubs from arid areas (Opuntia ficus-indica f. inermis and Atriplex nummularia L.) on growth and digestibility in lambs. Anim. Feed Sci. Technol. v.96, p.15–30. Doi: 10.1016/S0377-8401(01)00338-8 |
| 138 | Hadjipanayiotou, M. 2002. Replacement of soybean meal and barley grain by chickpeas in lamb and kid fattening diets. Anim. Feed Sci. Technol. v.96, p.103–109. Doi: 10.1016/S0377-8401(01)00339-X |
| 141 | Ravi, U., P. Singh, A.K. Garg, D.K. Agrawal. 2000. Performance of lambs fed expeller pressed and solvent extracted karanj (Pongamia pinnata) oil cake. Anim. Feed Sci. Technol. v.88, p.121–128. Doi: 10.1016/S0377-8401(00)00173-5 |
| 142 | Tolera, A., F. Sundstøl. 2000.Supplementation of graded levels of Desmodium intortum hay to sheep feeding on maize stover harvested at three stages of maturity: 1. Feed intake, digestibility and body weight change. Anim. Feed Sci. Technol. v.85, p.239–257. Doi: 10.1016/S0377-8401(00)00135-8 |
| 144 | Kaitho, R.J., N.N. Umunna, I.V. Nsahlai, S. Tamminga, J. Van Bruchem. 1998. Effect of feeding graded levels of Leucaena leucocephala, Leucaena pallida, Sesbania sesban and Chamaecytisus palmensis supplements to teff straw given to Ethiopian highland sheep. Anim. Feed Sci. Technol. v.72, p.355–366. Doi: 10.1016/S0377-8401(97)00159-4 |
| 147 | Al Jassim, R.A.M., F.T. Awadeh, A. Abodabos. 1997. Supplementary feeding value of urea-treated olive cake when fed to growing Awassi lambs. Anim. Feed Sci. Technol. v.64, p.287–292. Doi: 10.1016/S0377-8401(96)01044-9 |
| 148 | Bonsi, M.L.K., A.K. Tuah, P.O. Osuji, V.I. Nsahlai, N.N. Umunna. 1996. The effect of protein supplement source or supply pattern on the intake, digestibility, rumen kinetics, nitrogen utilisation and growth of Ethiopian Menz sheep fed teff straw. Anim. Feed Sci. Technol. v.64, p.11–25. Doi: 10.1016/S0377-8401(96)01048-6 |
| 149 | Nsahlai, I.V., N.N. Umunna. 1996. Sesbania and lablab supplementation of oat hay basal diet fed to sheep with or without maize grain. Anim. Feed Sci. Technol. v.61, p.275–289. Doi: 10.1016/0377-8401(95)00941-8 |
| 151 | Tuah, A.K., F.Y. Obese, E.R. Ørskov, D.B. Okai, D. Adomako, K.O. Amaning, A.N. Said, J.F.D. Greenhalgh. 1995. The performance of Djallonke sheep fed on diets containing various proportions of cocoa pod husk and 5% NaOH-treated maize cobs. Anim. Feed Sci. Technol. v.51, p.269–279. Doi: 10.1016/0377-8401(94)00690-B |
| 152 | Thi Hue, K., D.T.T. Van, I. Ledin, E. Spörndly, E. Wredle. 2010. Effect of feeding fresh, wilted and sun-dried foliage from cassava (Manihot esculenta Crantz) on the performance of lambs and their intake of hydrogen cyanide. Livest. Sci. v.131, p.155–161. Doi: 10.1016/j.livsci.2010.03.012 |
| 153 | Marie-Magdeleine, C., M. Boval, L. Philibert, A. Borde, H. Archimède. 2010. Effect of banana foliage (Musa x paradisiaca) on nutrition, parasite infection and growth of lambs. Livest. Sci. v.131, p.234–239. Doi: 10.1016/j.livsci.2010.04.006 |
| 155 | Nurfeta, A., A. Tolera, L.O. Eik, F. Sundstøl. 2009. Effect of enset (Ensete ventricosum) leaf supplementation on feed intake, digestibility, nitrogen utilization and body weight gain of sheep fed untreated or urea and calcium oxide-treated wheat straw. Livest. Sci. v.122, p.134–142. Doi: 10.1016/j.livsci.2008.08.005 |
| 156 | Manaye, T., A. Tolera, T. Zewdu. 2009. Feed intake, digestibility and body weight gain of sheep fed Napier grass mixed with different levels of Sesbania sesban. Livest. Sci. v.122, p.24–29. Doi: 10.1016/j.livsci.2008.07.020 |
| 157 | Mekoya, A., S.J. Oosting, S. Fernandez-Rivera, S. Tamminga, A. Tegegne, A.J. Van der Zijpp. 2009. Effect of supplementation of Sesbania sesban on post-weaning growth performance and sexual development of Menz sheep (Ethiopia). Livest. Sci. v.121, p.108–116. Doi: 10.1016/j.livsci.2008.06.012 |
| 158 | Nurfeta, A., A. Tolera, L.O. Eik, F. Sundstøl. 2008. The supplementary value of different parts of enset (Ensete ventricosum) to sheep fed wheat straw and Desmodium intortum hay. Livest. Sci. v.119, p.22–30. Doi: 10.1016/j.livsci.2008.02.010 |
| 161 | Assefa, G., C. Kijora, A. Kehaliew, S. Bediye, K.J. Peters. 2008. Evaluation of tagasaste (Chamaecytisus palmensis) forage as a substitute for concentrate in diets of sheep. Livest. Sci. v.114, p.296–304. Doi: 10.1016/j.livsci.2007.05.017 |
| 164 | Melaku, S., K.J. Peters, A. Tegegne. 2004. Effects of supplementation with foliages of selected multipurpose trees, their mixtures or wheat bran on feed intake, plasma enzyme activities, live weight and scrotal circumference gains in Menz sheep. Livest. Prod. Sci. v.89, p.253–264. Doi: 10.1016/j.livprodsci.2004.01.003 |
| 166 | Haddad, S.G., M.Q. Husein. 2004. Effect of dietary energy density on growth performance and slaughtering characteristics of fattening Awassi lambs. Livest. Prod. Sci. v.87, p.171–177. Doi: 10.1016/j.livprodsci.2003.09.018 |
| 167 | Nsahlai, I.V., H. Green, M. Bradford, M.L.K. Bonsi. 2002. The influence of source and level of protein, and implantation with zeranol on sheep growth. Livest. Prod. Sci. v.74, p.103–112. Doi: 10.1016/S0301-6226(01)00284-6 |
| 179 | Madruga, M.S., R.G. Costa, A.M. Silva, A.V.M.S. Marques, R.N. Cavalcanti, N. Narain, C.L.C. Albuquerque, G.E. Lira Filho. 2008. Effect of silk flower hay (Calotropis procera Sw) feeding on the physical and chemical quality of Longissimus dorsi muscle of Santa Inez lambs. Meat Sci. v.78, p.469–474. Doi: 10.1016/j.meatsci.2007.07.016 |
| 182 | Aguilera-Soto, J.I., R.G. Ramirez, C.F. Arechiga, F. Mendez-Llorente, M.A. Lopez-Carlos, J.M. Silva-Ramos, R.M. Rincon-Delgado, F.M. Duran-Roldan. 2008. Effect of Feed Additives in Growing Lambs Fed Diets Containing Wet Brewers Grains. Asian-australas. J. Anim. Sci. v.21, p.1425–1434. Doi: 10.5713/ajas.2008.70735 |
| 185 | Abu-Zanat., M.M.W. 2005. Voluntary Intake and Digestibility of Saltbush by Sheep. Asian-australas. J. Anim. Sci. v.18, p.214–220. Doi: 10.5713/ajas.2005.214 |
| 187 | Owaimer, A.N., M.S. Kraidees, M. Al-saiady, S. Zahran, M.A. Abouheif. 2004. Effect of Feeding Olive Cake in Complete Diet on Performance and Nutrient Utilization of Lambs. Asian-australas. J. Anim. Sci. v.17, p.491–496. Doi: 10.5713/ajas.2004.491 |
| 188 | Saiyed, L.H., S. Parnerkar, K.N. Wadhwani, P.R. Pandya, A.M. Patel. 2003. Performance of Weaner Lambs on Conventional Feeds or Supplemented with Mango Seed Kernel (Mangifera indica) and Babul Pods Chuni (Acacia nilotica) under Intensive Production System. Asian-australas. J. Anim. Sci. v.16, p.1469–1474. Doi: 10.5713/ajas.2003.1469 |
| 189 | Singh, N.P., S.K. Sankhyan, V.S.S. Prasad. 2003. Production Performance and Carcass Characteristics of Malpura and Mutton Synthetic Lambs Fed Low and High Energy Rations in a Semiarid Region of India. Asian-australas. J. Anim. Sci. v.16, p.655–659. Doi: 10.5713/ajas.2003.655 |
| 190 | Anigbogu, N.M. 2003. Supplementation of Dry Brewer's Grain to Lower Quality Forage Diet for Growing Lambs in Southeast Nigeria. Asian-australas. J. Anim. Sci. v.16, p.384–388. Doi: 10.5713/ajas.2003.384 |
| 191 | Nagalakshmi, D., V.R.B. Sastry, D.K. Agrawal. 2003. Relative Performance of Fattening Lambs on Raw and Processed Cottonseed Meal Incorporated Diets. Asian-australas. J. Anim. Sci. v.16, p.29–35. Doi: 10.5713/ajas.2003.29 |
| 193 | Singh, S., S.S. Kundu, A.S. Negi, S.K. Gupta, N.P. Singh, V.C. Pachouri. 2002. Leucaena Seeds as Protein Supplement in the Rations of Growing Sheep. Asian-australas. J. Anim. Sci. v.15, p.1433–1438. Doi: 10.5713/ajas.2002.1433 |
| 194 | El Hag, M.G., M.A. Al-Merza, B. Al Salti. 2002. Growth in the Sultanate of Oman of Small Ruminants Given Date Byproducts-Urea Multinutrient Blocks. Asian-australas. J. Anim. Sci. v.15, p.671–674. Doi: 10.5713/ajas.2002.671 |
| 195 | Rath, S., A.K. Verma, P. Singh, R.S. Dass, U.R. Mehra. 2001. Performance of Growing Lambs Fed Urea Ammoniated and Urea Supplemented Wheat Straw Based Diets. Asian-australas. J. Anim. Sci. v.14, p.1078–1084. Doi: 10.5713/ajas.2001.1078 |
| 198 | Khan, A.G., A. Azim, M. Nadeem. 1996. Effect of early weaning diets containing different protein sources on the performance of intensified Afghani Lambs. Asian-australas. J. Anim. Sci. v.9, p. 211–214. Doi: 10.5713/ajas.1996.211 |
| 205 | Koralagama, K.D.N., F.L. Mould, S. Fernandez-Rivera, J. Hanson. 2008. The effect of supplementing maize stover with cowpea (Vigna unguiculata) haulms on the intake and growth performance of Ethiopian sheep. Animal. v.2, p. 954–961. Doi: 10.1017/S1751731108001912 |
| 206 | Raghuvansi, S.K.S., R. Prasad, M.K. Tripathi, A.S. Mishra, O.H. Chaturvedi, A.K. Misra, B.L. Saraswat, R.C. Jakhmola. 2007. Effect of complete feed blocks or grazing and supplementation of lambs on performance, nutrient utilisation, rumen fermentation and rumen microbial enzymes. Animal. v.1, p. 221–226. Doi: 10.1017/S1751731107284058 |
| 208 | Eugène, M., H. Archimède, J.L. Weisbecker, F. Periacarpin, G. Saminadin, D. Sauvant. 2004. Effects of defaunation on digestion and growth, in sheep receiving a mixed diet (fresh Digitaria decumbens grass and concentrate) at four protein to energy ratios. Anim. Res. v.53, p. 111–125. Doi: 10.1051/animres:2004004 |
| 231 | Rihani, N., W.N. Garrett, R.A. Zinn. 1993. Effect of source of supplemental nitrogen on the utilization of citrus pulp-based diets by sheep. J. Anim. Sci. v.71, p.2310-2321. Doi: 10.2527/1993.7192310x |
| 252 | Bhattacharya, A.N., T.M. Khan, M. Uwayjan. 1975. Dried Beet Pulp as a Sole Source of Energy in Beef and Sheep Rations. J. Anim. Sci. v.41, p.616-621. Doi: 10.2527/jas1975.412616x |
| 253 | Bhattacharya, A.N., A.R. Khan. 1973. Wheat Straw and Urea in Pelleted Rations for Growing-Fattening Sheep. J. Anim. Sci. v.37, p.136-140. Doi: 10.2527/jas1973.371136x |
| 254 | Velloso, L., T.W. Perry, R.C. Peterson, W.M. Beeson. 1971. Effect of Dehydrated Alfalfa Meal and of Fish Solubles on Growth and Nitrogen and Energy Balance of Lambs and Beef Cattle Fed a High Urea Liquid Supplement. J. Anim. Sci. v.32, p.764-768. Doi: 10.2527/jas1971.324764x |
| 260 | Pailan, G.H., S.K. Mahanta, N.C. Verma, S.S. Kundu. 2007. Performance of sheep and goats maintained on rotational grazing with different levels of concentrate supplementation. Indian J. Anim. Sci. v.77, p.1161-1165. |
| 264 | Santra, A., S.A. Karim. 1999. Growth performance of weaner lambs maintained on varying levels of dietary protein in pre-weaning phase. Indian J. Anim. Sci. v.69, p.722-726. |
| 266 | Chauhan, J.S., M.C. Garg, M.P. Narang. 1997. Growth performance in Gaddi lambs. Indian J. Anim. Sci. v.67, p.244-245. |
| 267 | Reddy, K.J.; M.R. Reddy. 1985. Effect of feeding complete rations on growth performance and nutrient utilization in nellore sheep. Indian J. Anim. Sci. v.55, p.459-463. |
| 269 | Kuldip., M.A. Akbar, A.B. Mandal, P.C. Gupta. 1995. Comparative feeding value of sunflower and mustard-cake in sheep. Indian J. Anim. Sci. v.65, p.944-946. |
| 270 | Kuldip., M.A. Akbar, A.B. Mandal, P.C. Gupta. 1995. Nutritional evaluation of sunflower-cake in sheep. Indian J. Anim. Sci. v.65, p.820-823. |
| 272 | Din, E., A.R. Tag. 1992. Utilization of water-hyacinth hay in feeding of growing sheep. Indian J. Anim. Sci. v.62, p.989-992. |
| 274 | Krishna Mohan, D.V.G., R.K. Krishna, M.A. Srirama. 1987. Protein requirements of crossbred lambs. Indian J. Anim. Sci. v.57, p.1121-1127. |
| 275 | Krishna Mohain, D.V.G., C.M. Naidu, K.K. Reddy. 1985. Feedlot performance of Mandya and Nellore cross lambs. Indian J. Anim. Sci. v.55, p.1087-1091. |
| 277 | Prasad, R.D.D., D.A. Prasad, R.R. Reddy. 1986. Evaluation of complete rations containing groundnut haulms for lambs. Indian J. Anim. Sci. v.56, p.258-261. |
| 278 | Singh, N.P. 1985. Growth and nutrient utilization in crossbred lambs fed on lucerne and cowpea hay-meal complete rations. Indian J. Anim. Sci. v.55, p.715-716. |
| 279 | Purushotham, N.P., M.S. Rao, G.V. Raghavan. 1986. Utilization of castor beanmeal in the concentrate mixture of sheep. Indian J. Anim. Sci. v.56, p.1090-1093. |
| 280 | Lall, D.; J. Kishan, S.S. Negi. 1984. Feeding value of dried rabbit excreta in the ration of sheep. Indian J. Anim. Sci. v.54, p.1005-1007. |
| 286 | Singh, N.P., B.C. Patnayak. 1980. Urea in the concentrate supplement for cross bred weaner lambs. Indian J. Anim. Sci. v.50, p. 496-498. |
| 288 | Nath, K., V.K. Vijjan, G. Krishna, S.K. Ranjhan. 1979. Relative value of single-cell proteins (SCP) and groundnut-cake as a protein supplement in the ration of lambs. Indian J. Anim. Sci. v.49, p. 199-202. |
| 291 | Anigbogu, N.M., P. Bienstman, B. Van Damme, C.D. Ezeokoli. 2006. Incorporation of Dry Mangifera indica Kernel in the Concentrate Ration of Growing Lambs. Revue Élev. Méd. vét. Pays trop. v.59, p.39-42. Doi: 10.19182/remvt.9952 |
| 292 | Ayantunde, A.A., S. Fernandez Rivera, A. Dan-Gomma. 2008. Sheep Fattening with Groundnut Haulms and Millet Bran in the West African Sahel. Revue Élev. Méd. vét. Pays trop. v.61, p.215-220. Doi: 10.19182/remvt.9992 |
| 294 | Sayed, A.B.N. 2009. Effect of Different Dietary Energy Levels on the Performance and Nutrient Digestibility of Lambs. Vet. World. v.2, p.418-420. |
| 295 | Hosseini, S.M., S.M. Akbary, N. Maheri-Sis, A.M. Aghsaghali. 2008. Effect of Different Energy Levels of Diet on Feed Efficiency, Growth Rate and Carcass Characteristics of Fattening Bahmaei Lambs. J. Anim. Vet. Adv. v.7, p.1551-1554. |
| 300 | Bahrami, Y., A.D. Foroozandeh, F. Zamani, M. Modarresi, S. Eghbal-Saeid, S. Chekani-Azar. 2010. Effect of diet with varying levels of dried grape pomace on dry matter digestibility and growth performance of male lambs. J. Anim. Plant Sci. v.6, p.605-610. |
| 302 | Jabbar, M.A., M.I. Anjum. 2008. Effect of diets with different forage to concentrate ratio for fattening of Lohi lambs. Pakistan Vet. J., v.28, p.150-152. |
| 303 | Einkamerer, O.B., H.O. de Waal, W.J. Combrinck, M.D. Fair. 2009. Feed utilization and growth of Dorper wethers on Opuntia-based diets. S. Afr. J. Anim. Sci. v.39, p.53-57. |
| 304 | Rihani, N., F.Guessous, A. Berrami. 1988. Utilisation de quelques sous-produits de l’agro-industrie pourl engraissement des ovins. Communication présentée aux 18 Journées de lº ANPA en Mars 1988. Ces études ont été réalisées dans lecadres: “petits Ruminants” (SR-CRSP). |
| 305 | Guessous, F., A. El Hilali, W.L. Johnson. 1988. Influence du taux d’incorporation de la pulpe de caroube sur la digestibilité et l’utilisation des rations par des ovins à l’engraissement. Reprod. Nutr. v.28, p.93-94. |
| 307 | Darawish, S., M.Y. Harb, H.H. Titi. 2010. Effects of Replacing Alfalfa Hay by Barley Straws on Growth Performance, Carcass Characteristics and Nutrients Digestibility for Fattening Awassi Lambs. Jordan J. Agric. Sci. v.6, p.27-39. |
| 308 | Canbolat, O., A. Karabulut. 2010. Effect of urea and oregano oil supplementation on growth performance and carcass characteristics of lamb fed diets containing different amounts of energy and protein. Turkish J. Vet. Anim. Sci. v.34, p. 119-128. Doi: 10.3906/vet-0708-20 |
| 310 | Nkosi, B.D., R. Meeske, H.J. van der Merwe, O. Acheampong-Boateng, T. Langa. 2010. Effects of dietary replacement of maize grain with popcorn waste products on nutrient digestibility and performance by lambs. S. Afr. J. Anim. Sci. v.40, p.133-139. |
| 315 | El-Baki, S.A., E. Soliman, E. Abou-Fandoud, A. Aiad. 2005. Clays in animal nutrition: 6. Effect of feeding pelleted complete feeds contained urea and tafla on digestibility, growth and wool of local sheep. Egypt . J. Sheep and Goats Sci. Doi: 10.21608/EJSGS.2005.28471 |
| 316 | Tajori, R.M. 2009. Evaluation of use of olive cake silage in sheep feeding and its affect on growth rate. Proceedings of the 2^nd^ Scientific Conference of animal wealth research in the Middle East and North Africa, Cairo, International Convention Center, October 24-26, pp.441-449. |
| 317 | Alem, M., B. Tamir, M.Y. Kurtu. 2011. Feed utilisation of Ethiopian Highland lambs on a basal diet of Eleucine coracana straw and supplemented with variously sourced protein mixed with wheat bran. Trop. Anim. Health Prod. v.43, p.115-120. Doi: 10.1007/s11250-010-9662-7 |
| 320 | Soodeen-Karamath, S. F.G. Youssef. 1999. Effect of monensin, avoparcin and grass supplementation on utilization of urea-treated rice straw by sheep and goats. Small Rumin. Res. v.33, p.201-211. Doi: 10.1016/S0921-4488(99)00031-0 |
| 325 | Dada, S.A.O., J.A. Adeneye, A.O. Akinsoyinu, J.W. Smith, K.E. Dashiell. 1999. Performance of sheep fed soybean stover and cassava crumb based diets. Small Rumin. Res. v.31, p.229-238. Doi: 10.1016/S0921-4488(98)00143-6 |
| 327 | Gionbelli, T.R.S., C.M. Veloso, M.P. Gionbelli, M.A.S. Novais, A.L. Silva, C.J.B. Espechit, J.M.S. Campos, S.C. Valadares Filho, O.G. Pereira, C.S. Cunha, P.H. Alcântara, G.F. Virgínio Junior, M.S. Duarte. 2014. Utilization of castor bean meal treated with calcium hydroxide, fed wet or dry, by lambs. Livest. Sci., v.168, p.76-83. Doi: 10.1016/j.livsci.2014.08.012 |
| 328 | S.A. Hassan, R.A.M. Al Jassim, A.N. Al-Ani, N.S. Abdullah. 1991. Effects of dietary supplement of rumen undegradable protein upon carcass composition of fat-tail Awassi sheep. Small Rumin. Res. v.5, p.65-74. Doi: 10.1016/0921-4488(91)90031-K |
| 329 | Haddad, S.G., B.S. Obeidat. 2007. Effects of dietary supplement of rumen undegradable protein upon carcass composition of fat-tail Awassi sheep. Small Rumin. Res. v.69, p.23-27. Doi: 10.1016/j.smallrumres.2005.12.004 |
| 333 | Costa, M.R.G.F., E.S. Pereira, A.M.A. Silva, P.V.R. Paulino, I.Y. Mizubuti, P.G. Pimentel, A.P. Pinto, J.N. Rocha Junior. 2013. Body composition and net energy and protein requirements of Morada Nova lambs. Small Rumin. Res. v.114, p.206-213. Doi: 10.1016/j.smallrumres.2013.06.014 |
| 334 | Galvani, D.B., A.V. Pires, I. Susin, V.N. Gouvêa, A. Berndt, L.J. Chagas, J.R.R. Dórea, A.L. Abdalla, L.O. Tedeschi. 2014. Energy efficiency of growing ram lambs fed concentrate-based diets with different roughage sources. J. Anim. Sci. v.92, p.250-263. Doi: 10.2527/jas.2012-6017 |
| 335 | Pereira, E.S., R.M. Fontenele, A.M.A. Silva, R.L. Oliveira, M.R.G. Ferreira, I.Y. Mizubuti, M.S.S. Carneiro & A.C.N. Campos. Body Composition and Net Energy Requirements of Brazilian Somali Lambs. Ital. J. Anim. Sci. v.13, p.880-886. Doi: 10.4081/ijas.2014.3583 |
| 337 | Barbera, M., J.R. Jaber, S. Ahmed-Salek, A. Ravelo-Garcia, E. Rodríguez-Ponce, L. Rey, M.R. Ventura. 2018. Effects of replacing rye-grass (Lolium spp.) hay by banana (Musa acuminata L.) by-products on feed intake, growth, and feed conversion rate of Canary hair sheep breed (Pelibuey) lambs. Trop. Anim. Health Prod. v.50, p.1941-1945. Doi: 10.1007/s11250-018-1618-3 |
| 339 | Regadas Filho, J.G.L., E.S. Pereira, P.G. Pimentel, A.B.S. Villarroel, A.N. Medeiros, R.M. Fontenele. 2013. Body composition and net energy requirements for Santa Ines lambs. Small Rumin. Res. v.109, p.107-112. Doi: 10.1016/j.smallrumres.2012.07.011 |
| 340 | Alemu, D., Tegegne, F., Mekuriaw, Y. 2020. Comparative evaluation of effective microbe- and urea molasses-treated finger millet (Eleusine coracana) straw on nutritive values and growth performance of Washera sheep in northwestern Ethiopia. Trop. Anim. Health Prod. v.52, p.123-129. Doi: 10.1007/s11250-019-01986-z |
| 344 | E.S. Pereira, F.W.R. Lima, M.I. Marcondes, J.P.P. Rodrigues, A.C.N. Campos, L.P. Silva, L.R. Bezerra, M.W.F. Pereira, R.L. 2017. Oliveira. Energy and protein requirements of Santa Ines lambs, a breed of hair sheep. Animal. v.11, p.2165-2174. Doi: 10.1017/S1751731117001185 |
| 345 | Candyrine, S.C.L., M.F. Jahromi, M. Ebrahimi, W.L. Chen, S. Rezaei, Y.M. Goh, N. Abdullah, J.B. Liang. 2019. Oil supplementation improved growth and diet digestibility in goats and sheep fed fattening diet. Asian-Australas. J. Anim. Sci. v.32, p.533-540. Doi: 10.5713/ajas.18.0059 |
| 346 | Leite, P.M.B.A., R.M.L. Véras, A.S.C. Véras, A. Guim, E.J.O. Souza, K.K.S. Silva, L.M.G. Barreto, J.L. Silva & D.B. Cardoso. Different roughage:concentrate ratios with and without liquid residue of cassava for lambs. Trop. Anim. Health Prod. v.50, p.1807-1814. Doi: 10.1007/s11250-018-1622-7 |
| 347 | Dos Santos, A.C.S., S.A. Santos, G.G.P. Carvalho, L.D.S. Mariz, M.SL. Tosto, S.C. Valadares Filho, J.A.G. Azevedo. 2018. A comparative study on the excretion of urinary metabolites in goats and sheep to evaluate spot sampling applied to protein nutrition trials. J. Anim. Sci. v.96, p. 3381-3397. Doi: 10.1093/jas/sky198 |
| 348 | Negewo, T., S. Melaku, B. Asmare, A. Tolera. 2018. Performance of Arsi-Bale sheep fed urea treated maize cob as basal diet and supplemented with graded levels of concentrate mixture. Trop. Anim. Health Prod. v.50, p.1209-1217. Doi: 10.1007/s11250-018-1544-4 |
| 349 | Camilo, F.R., F.M. Vargas Junior, H.A. Ricardo, A.R.M. Fernandes, L.O. Seno, J.C.S. Osório, M.R. Souza, A.M. Mobiglia. 2015. The intake of thermally processed soybean reduces the feedlot period of lambs independently of roughage to concentrate ratio. J. Anim. Sci. v.93, p. 3084-3094. Doi: 10.2527/jas.2014-8560 |
| 351 | Dávila-Ramírez, J.L., U. Macías-Cruz, N.G. Torrentera-Olivera, H. González-Ríos, E.A. Peña-Ramos, S.A. Soto-Navarro, L. Avendaño-Reyes. 2015. Feedlot performance and carcass traits of hairbreed ewe lambs in response to zilpaterol hydrochloride and soybean oil supplementation. J. Anim. Sci. v.93, p. 3189-3196. Doi: 10.2527/jas.2014-8723 |
| 352 | Rodrigues, R.T.S., M.L. Chizzotti, S.R. Martins, I.F. Silva, M.A.Á. Queiroz, T.S. Silva, K.C. Busato, A.M.A. Silva. Energy and protein requirements of non-descript breed hair lambs of different sex classes in the semiarid region of Brazil. Trop. Anim. Health Prod. v.48, p.87-94. Doi: 10.1007/s11250-015-0924-2 |
| 357 | Sudarman, A., M. Hayashida, I.R. Puspitaning, A. Jayanegara, H. Shiwachi. 2016. The use of cassava leaf silage as a substitute for concentrate feed in sheep. Trop. Anim. Health Prod. v.48, p.1509-1512. Doi: 10.1007/s11250-016-1107-5 |
| 358 | Costa, J.B., R.L. Oliveira, T.M. Silva, R.D.X. Ribeiro, A.M. Silva, A.G. Leão, L.R. Bezerra, T.C. Rocha. 2016. Intake, digestibility, nitrogen balance, performance, and carcass yield of lambs fed licuri cake. J. Anim. Sci. v.94, p. 2973-2980. Doi: 10.2527/jas.2015-0143 |
| 361 | Bhatt, R.S., A. Sahoo. 2017. Effect of feeding complete feed block containing rumen protected protein, non-protein nitrogen and rumen protected fat on improving body condition and carcass traits of cull ewes. J. Anim. Physiol. Anim. Nutr. v.101, p. 1147-1158. Doi: 10.1111/jpn.12628 |
| 362 | Alpízar-Naranjo, A., J. Arece-García, M. Esperance, Y. López, M. Molina, E. González-García. 2017. Partial or total replacement of commercial concentrate with on-farm-grown mulberry forage: effects on lamb growth and feeding costs. Trop. Anim. Health Prod. v.49, p.537-546. Doi: 10.1007/s11250-017-1225-8 |
| 364 | Tadayon, Z., Y. Rouzbehan, J. Rezaei. 2017. Effects of feeding different levels of dried orange pulp and recycled poultry bedding on the performance of fattening lambs. J. Anim. Sci. v.95, p. 1751-1765. Doi: 10.2527/jas.2016.0889 |
| 365 | Kumar, R., S.K. Saha, D. Kumar, M.S. Mahesh, C.D. Malapure. 2017. Effect of dietary utilisation of sugarcane press mud on production performance of Muzaffarnagari lambs. Trop. Anim. Health Prod. v.49, p.1439-1446. Doi: 10.1007/s11250-017-1345-1 |
| 367 | Mirmohammadi, D., Y. Rouzbehan, H. Fazaeli. 2015. The effect of the inclusion of recycled poultry bedding and the physical form of diet on the performance, ruminal fermentation, and plasma metabolites of fattening lambs. J. Anim. Sci. v.93, p. 3843-3853. Doi: 10.2527/jas.2014-8789 |
| 368 | Imani Rad, M., Y. Rouzbehan, J. Rezaei. 2016. Effect of dietary replacement of alfalfa with urea-treated almond hulls on intake, growth, digestibility, microbial nitrogen, nitrogen retention, ruminal fermentation, and blood parameters in fattening lambs. J. Anim. Sci. v.94, p. 349-358. Doi: 10.2527/jas.2015-9437 |

^1^Publication ID refers to the publication code for each study in the database.

**Table S2.** Studies from the goat dataset used for model development and evaluation.

| **Publication ID^1^** | **Reference** |
| --- | --- |
| 373 | Singh, S., S.S. Kundu, A.S. Negi, V.C. Pachouri. 2010. Performance of growing kids on rations with Lablab (Lablab purpureus) grains as protein source. Livest. Res. Rural. Dev. v.22. |
| 374 | Abdulrazak, S.A., E.G. Njuguna, P.K. Karau. 2005. The effect of supplementing Rhodes grass (Chloris gayana) hay with Acacia tortilis leaves and pods mixture on intake, digestibility and growth performance of goats. Livest. Res. Rural. Dev. v.17. |
| 376 | Atti, N., H. Rouissi, M. Mahouachi. 2004. The effect of dietary crude protein level on growth, carcass and meat composition of male goat kids in Tunisia. Small Rumin. Res. v.54, p.89-97. Doi: 10.1016/j.smallrumres.2003.09.010 |
| 378 | Phengvichith, V., I. Ledin. 2007. Effect of a diet high in energy and protein on growth, carcase characteristics and parasite resistance in goats. Trop. Anim. Health Prod. v.39, p.59-70. Doi: 10.1007/s11250-006-4443-z |
| 379 | Sheridan, R., A.V Ferreira, L.C. Hoffman. 2003. Production efficiency of South African Mutton Merino lambs and Boer goat kids receiving either a low or a high energy feedlot diet. Small Rumin. Res. v.50, p.75-82. Doi: 10.1016/S0921-4488(03)00109-3 |
| 380 | Mahgoub, O., C.D. Lu, M.S. Hameed, A. Richie, A.S. Al-Halhali, K Annamalai. 2003. Performance of Omani goats fed diets containing various metabolizable energy densities. Small Rumin. Res. v.58, p.175-180. Doi: 10.1016/j.smallrumres.2004.09.008 |
| 382 | El Shaer, H., H.M. Kandil, H.S. Khamis, H.M. Abou El-Nasr. 1997. Alternative feed supplement resources for sheep and goats in Egypt. In: Lindberg J.E. (ed.), Gon da H.L. (ed.), Ledin I. (ed.). Recent advances in small ruminant nutrition. Zaragoza: CIHEAM, p. 93-97 (Options Méditerranéennes: Série A. Séminaires Méditerranéens; n. 34) |
| 383 | Phimphachanhvongsod, V., Ledin, I. 2002. Performance of Growing Goats Fed Panicum maximum and Leaves of Gliricidia sepium. Asian-australas. J. Anim. Sci. v.15, p.1585–1590. Doi: 10.5713/ajas.2002.1585 |
| 384 | Ondiek, J.O., S.A. Abdulrazak, J.K. Tuitoek, F.B. Bareeba. 1999. The effects of Gliricidia sepium and maize bran as supplementary feed to Rhodes grass hay on intake, digestion and liveweight of dairy goats. Livest. Prod. Sci. v.61, p.65–70. Doi: 10.1016/S0301-6226(99)00004-4 |
| 387 | Phengvichith, V., I. Ledin. 2007. Effects of Supplementing Gamba Grass (Andropogon gayanus) with Cassava (Manihot esculenta Crantz) Hay and Cassava Root Chips on Feed Intake, Digestibility and Growth in Goats. Asian-australas. J. Anim. Sci. v.20, p.725–732. Doi: 10.5713/ajas.2007.725 |
| 388 | Islam, M., S.A. Chowdhury, M. R. Alam. 1997. The effect of supplementation of jackfruit leaves (Artocarpus heterophyllus) and mashkalai (Vigna mungo) bran to common grass on the performance of goats. Asian-Aust. J. Anim. Sci. v.10, p.206-209. Doi: 10.5713/ajas.1997.206 |
| 389 | Ifut, O. J., 1992. Body weight response of West African Dwarf goats fed Gliricidia sepium, Panicum maximum and cassava (Manihot) peels. Stares J E S. Said AN and Kategile J A (eds). 1992. The complementarity of feed resources for animal production in Africa. Proc. Joint feed resources networks workshop held in Gaborone, Botswana 4-8 March 1991. African Feeds Research Network. |
| 390 | Yousuf, M.B., M.A. Belewu, J.O. Daramola, N.I. Ogundun. 2007. Protein supplementary values of cassava, leucaena and gliricidia leaf meals in goats fed low quality Panicum maximum hay. Livest. Res. Rural. Dev. v.19. |
| 391 | Aregheore, E.M. 2007. Voluntary intake, nutrient digestibility and nutritive value of foliage of fluted pumpkin (Talfairia occidentialis) - haylage mixtures by goats. Livest. Res. Rural. Dev. v.19. |
| 392 | Atti, N., M. Mahouachi, F. Zouaghi, H. Rouissi. 2009. Incorporation of cactus (Opuntia ficus-indica f. inermis) in young goats diets: 1. Effects on intake, digestion, growth and carcass composition. Livest. Res. Rural. Dev. v.21. |
| 393-394 | Aregheore, E.M., D. Perera, M.S. Yahaya. 2004. Nutritive Value of Batiki Grass (Ischaemum aristatum var. indicum) Supplemented with Leaves of Browses (Gliricidia sepium and Leucaena leucocephala) on Performance of Goats. Int. J. Agric. Biol. v.6, p.143-148. |
| 395 | Chobtang, J., K. Intharak, A. Isuwan. 2009. Effects of dietary crude protein levels on nutrient digestibility and growth performance of Thai indigenous male goats. Songklanakarin J. Sci. Technol. v.31, p.591-596. |
| 396 | Hango, A., L.A. Mtenga, G.C. Kifaro, J. Safari, D.E. Mushi, V.R.M. Muhikambele. 2007. A study on growth performance and carcass characteristics of Small East African goats under different feeding regimes. Livest. Res. Rural. Dev. v.19. |
| 397 | Kinuthia, M. N., M.M. Wanyoike, C.K. Gachuiri, J.W. Wakhungu. 2007. Effect of supplementing weaner goats with graded levels of Calliandra calothyrsus and Lucerne (Medicago sativa) on feed intake and weight gain. Livest. Res. Rural. Dev. v.19. |
| 401 | Ngi, J., J.A. Ayoade, O.I.A. Oluremi. 2006. Evaluation of dried cassava leaf meal and maize offal as supplements for goats fed rice straw in dry season. Livest. Res. Rural. Dev. v.18. |
| 403 | Wambui, C.C., S.A. Abdulrazak, and Q Noordin. 2006. The effect of supplementing urea treated maize stover with Tithonia, Calliandra and Sesbania to growing goats. Livest. Res. Rural. Dev. v.18. |
| 405 | Osakwe, I.I., R.N. Udeogu. 2007. Feed intake and nutrient digestibility of west African dwarf (WAD) goat fed Pennisetum purpureum supplemented with Gmelina arborea. Anim. Res. Int. v.4, p.724-727. |
| 407 | Sawe, J.J., J.K. Tuitoek, J.M. Ottaro. 1998. Evaluation of common tree leaves or pods as supplements for goats on range area of Kenya. Small Rumin. Res. v.28, p.31-37. Doi: 10.1016/S0921-4488(97)00059-X |
| 413 | Kaitho, R.J., A. Tegegne, N.N. Umunna, I.V. Nsahlai, S. Tamminga, J. Van Bruchem, J.M. Arts. 1998. Effect of Leucaena and Sesbania supplementation on body growth and scrotal circumference of Ethiopian highland sheep and goats fed teff straw basal diet. Livest. prod. sci. v.54, p.173-181. Doi: 10.1016/S0301-6226(97)00152-8 |
| 414 | Alemu, W., S. Melaku, A. Tolera. 2010. Supplementation of cottonseed, linseed, and noug seed cakes on feed intake, digestibility, body weight, and carcass parameters of Sidama goats. Trop. Anim. Health Prod. v.42, p.623-631. Doi: 10.1007/s11250-009-9466-9 |
| 417 | Aregheore, E.M. 2001. Growth rate, apparent nutrient digestibility and some blood metabolites of Gwembe valley goats on rations based on crop residues in the hot dry season in Zambia. Trop. Anim. Health Prod. v.33, p.331-340. Doi: 10.1023/a:1010592104189 |
| 418 | Mupangwa, J.F., N.T. Ngongoni, J.H. Topps, H. Hamudikuwanda. 2000. Effects of supplementing a basal diet of Chloris gayana hay with one of three protein-rich legume hays of Cassia rotundifolia, Lablab purpureus and Macroptilium atropurpureum forage on some nutritional parameters in goats. Trop. Anim. Health Prod. v.32, p.245-256. Doi: 10.1023/a:1005283603781 |
| 420 | Pal, A., R.K. Sharma, R. Kumar, K. Barman. 2010. Effect of replacement of concentrate mixture with isonitrogenous leaf meal mixture on growth, nutrient utilization and rumen fermentation in goats. Small Rumin. Res. v.91, p.132-140. Doi: 10.1016/j.smallrumres.2010.02.012 |
| 421 | Dutta, T.K., M.K. Agnihotri, P.K. Sahoo, V. Rajkumar, A.K. Das. 2009. Effect of different protein–energy ratio in pulse by-products and residue based pelleted feeds on growth, rumen fermentation, carcass and sausage quality in Barbari kids. Small Rumin. Res. v.85, p.34-41. Doi: 10.1016/j.smallrumres.2009.07.002 |
| 423 | Phengvichith, V., I. Ledin. 2007. Effect of feeding different levels of wilted cassava foliage (Manihot esculenta, Crantz) on the performance of growing goats. Small Rumin. Res. v.71, p.109-116. Doi: 10.1016/j.smallrumres.2006.05.009 |
| 424 | Costa, R.G., M.X.C. Correia, J.H.V. Da Silva, A.N. De Medeiros, F.F.R. De Carvalho. 2007. Effect of different levels of dehydrated pineapple by-products on intake, digestibility and performance of growing goats. Small Rumin. Res. v.71, p.138-143. Doi: 10.1016/j.smallrumres.2006.05.012 |
| 425 | Kahindi, R.K., S.A. Abdulrazak, R.W. Muinga. 2007. Effect of supplementing Napier grass (Pennisetum purpureum) with Madras thorn (Pithecellobium dulce) on intake, digestibility and live weight gains of growing goats. Small Rumin. Res. v.69, p.83-87. Doi: 10.1016/j.smallrumres.2005.12.008 |
| 427 | Bhatta, R., S. Vaithiyanathan, N.P. Singh, D.L. Verma. 2007. Effect of feeding complete diets containing graded levels of Prosopis cineraria leaves on feed intake, nutrient utilization and rumen fermentation in lambs and kids. Small Rumin. Res. v.67, p.75–83. Doi: 10.1016/j.smallrumres.2005.09.027 |
| 430 | Aregheore, E.M. 2006. Utilization of concentrate supplements containing varying levels of copra cake (Cocos nucifera) by growing goats fed a basal diet of napier grass (Pennisetum purpureum). Small Rumin. Res. v.64, p.87–93. Doi: 10.1016/j.smallrumres.2005.04.003 |
| 433 | Haddad, S.G. 2005. Effect of dietary forage:concentrate ratio on growth performance and carcass characteristics of growing Baladi kids. Small Rumin. Res. v.57, p.43–49. Doi: 10.1016/j.smallrumres.2004.05.001 |
| 436 | M.A Galina, M. Guerrero, C. Puga, G.F.W. Haenlein. 2004. Effect of a slow-intake urea supplementation on growing kids fed corn stubble or alfalfa with a balanced concentrate. Small Rumin. Res. v.53, p.29–38. Doi: 10.1016/j.smallrumres.2003.08.009 |
| 437 | Aregheore, E.M. 2004. Nutritive value of sweet potato (Ipomea batatas (L) Lam) forage as goat feed: voluntary intake, growth and digestibility of mixed rations of sweet potato and batiki grass (Ischaemum aristatum var. indicum). Small Rumin. Res. v.51, p.235–241. Doi: 10.1016/S0921-4488(03)00198-6 |
| 438 | Anbarasu, C., N. Dutta, K. Sharma, M. Rawat. 2004. Response of goats to partial replacement of dietary protein by a leaf meal mixture containing Leucaena leucocephala, Morus alba and Tectona grandis. Small Rumin. Res. v.51, p.47–56. Doi: 10.1016/S0921-4488(03)00203-7 |
| 439 | Soto-Navarro, S.A., A.L. Goetsch, T. Sahlu, R. Puchala. 2004. Effects of level and source of supplemental protein in a concentrate-based diet on sites of digestion and small intestinal amino acid disappearance in Boer × Spanish wether goats. Small Rumin. Res. v.51, p.101–106. Doi: 10.1016/S0921-4488(03)00187-1 |
| 440 | Titi, H.H. 2003. Replacing soybean meal with sunflower meal with or without fibrolytic enzymes in fattening diets of goat kids. Small Rumin. Res. v.48, p.45–50. Doi: 10.1016/S0921-4488(03)00003-8 |
| 441 | Bueno, M.S., E. Ferrari Jr., D. Bianchini, F.F. Leinz, C.F.C. Rodrigues. 2002. Effect of replacing corn with dehydrated citrus pulp in diets of growing kids. Small Rumin. Res. v.46, p.179–185. Doi: 10.1016/S0921-4488(02)00184-0 |
| 443 | Bamikole, M.A., I. Ezenwa, A.O. Akinsoyinu, M.O. Arigbede, O.J. Babayemi. 2001. Performance of West African dwarf goats fed Guinea grass–Verano stylo mixture, N-fertilized and unfertilized Guinea grass. Small Rumin. Res. v.39, p.145–152. Doi: 10.1016/S0921-4488(00)00182-6 |
| 446 | Singh, P., A.K. Verma, R.S. Dass, U.R. Mehra. 1999. Performance of pashmina kid goats fed oak (Quercus semecarpifolia) leaves supplemented with a urea molasses mineral block. Small Rumin. Res. v.31, p.239–244. Doi: 10.1016/S0921-4488(98)00142-4 |
| 447 | Srivastava, S.N.L., K. Sharma. 1998. Response of goats to pelleted diets containing different proportions of sun-dried Leucaena leucocephala. Small Rumin. Res. v.28, p.139–148. Doi: 10.1016/S0921-4488(97)00069-2 |
| 450 | Anandan, S., V.R.B. Sastry, L.M. Musalia, D.K. Agrawal. 1996. Growth rate and nutrient efficiency of growing goats fed urea ammoniated neem (Azadirachta indica) seed kernel meal as protein supplement. Small Rumin. Res. v.22, p.205–212. Doi: 10.1016/S0921-4488(96)00889-9 |
| 451 | Singh, P., J.C. Biswas, R. Somvanshi, A.K. Verma, S.M. Deb, R.A. Dey. 1996. Performance of pashmina (Cheghu) goats fed on oak (Quercus semecarpifolia) leaves. Small Rumin. Res. v.22, p.123–130. Doi: 10.1016/S0921-4488(96)00870-X |
| 452 | Chandrasekharaiah, M., M.R. Reddy, G.V.N. Reddy. 1996. Effect of feeding urea treated maize stover on growth and nutrient utilization by sheep and goats. Small Rumin. Res. v.22, p.141–147. Doi: 10.1016/S0921-4488(96)00864-4 |
| 454 | Saikia, G., K.K. Baruah, S.C. Buragohain, B.N. Saikia, N.N. Pathak. 1995. Feed intake, utilization of nutrients and growth of Assamese × Beetal goats fed three levels of energy. Small Rumin. Res. v.15, p.279–282. Doi: 10.1016/0921-4488(94)00025-3 |
| 455 | Verma, A.K., V.R.B. Sastry, D.K. Agrawal. 1995. Feeding of water washed neem (Azadirachta indica) seed kernel cake to growing goats. Small Rumin. Res. v.15, p.105–111. Doi: 10.1016/0921-4488(94)00018-3 |
| 456 | D.M. Zamudio, J.M. Pinos-Rodríguez, S.S. González, P.H. Robinson, J.C. García, O. Montañez. 2009. Effects of Agave salmiana Otto Ex Salm-Dyck silage as forage on ruminal fermentation and growth in goats. Anim. Feed Sci. Technol. v.148, p.1–11. Doi: 10.1016/j.anifeedsci.2008.02.002 |
| 458 | Hadjipanayiotou, M. 2002. Replacement of soybean meal and barley grain by chickpeas in lamb and kid fattening diets. Anim. Feed Sci. Technol. v.96, p.103–109. Doi: 10.1016/S0377-8401(01)00339-X |
| 459 | Nherera, F.V., L.R. Ndlovu, B.H. Dzowela. 1998. Utilisation of Leucaena diversifolia, Leucaena esculenta, Leucaena pallida and Calliandra calothyrsus as nitrogen supplements for growing goats fed maize stover. Anim. Feed Sci. Technol. v.74, p.15–28. Doi: 10.1016/S0377-8401(98)00164-3 |
| 460 | Oni, A.O., O.M. Arigbede, O.O. Oni, C.F.I. Onwuka, U.Y. Anele, B.O. Oduguwa, K.O. Yusuf. 2010. Effects of feeding different levels of dried cassava leaves (Manihot esculenta, Crantz) based concentrates with Panicum maximum basal on the performance of growing West African Dwarf goats. Livest. Sci., v.129, p.24-30. Doi: 10.1016/j.livsci.2009.12.007 |
| 461 | J. Safari, D.E. Mushi, L.A. Mtenga, G.C. Kifaro, L.O. Eik. 2009. Effects of concentrate supplementation on carcass and meat quality attributes of feedlot finished Small East African goats. Livest. Sci., v.125, p.266-274. Doi: 10.1016/j.livsci.2009.05.007 |
| 462 | Mushi, D.E., J. Safari, L.A. Mtenga, G.C. Kifaro, L.O. Eik. 2009. Effects of concentrate levels on fattening performance, carcass and meat quality attributes of Small East African × Norwegian crossbred goats fed low quality grass hay. Livest. Sci., v.124, p.148-155. Doi: 10.1016/j.livsci.2009.01.012 |
| 464 | Solomon, M., S. Melaku, A. Tolera. 2008. Supplementation of cottonseed meal on feed intake, digestibility, live weight and carcass parameters of Sidama goats. Livest. Sci., v.119, p.137-144. Doi: 10.1016/j.livsci.2008.03.011 |
| 465 | Oni, A.O., C.F.I. Onwuka, O.O. Oduguwa, O.S. Onifade, O.M. Arigbede. 2008. Utilization of citrus pulp based diets and Enterolobium cyclocarpum (JACQ. GRISEB) foliage by West African dwarf goats. Livest. Sci., v.117, p.184-191. Doi: 10.1016/j.livsci.2007.12.010 |
| 467 | Mui, N.T., I. Ledin, P. Udén, D.V. Binh. 2001. Effect of replacing a rice bran–soya bean concentrate with Jackfruit (Artocarpus heterophyllus) or Flemingia (Flemingia macrophylla) foliage on the performance of growing goats. Livest. Prod. Sci., v.72, p.253-262. Doi: 10.1016/S0301-6226(01)00223-8 |
| 471 | Paengkoum, P., J.B. Liang, Z.A. Jelan, M. Basery. 2006. Utilization of Steam-treated Oil Palm Fronds in Growing Saanen Goats: II. Supplementation with Energy and Urea. Asian-australas. J. Anim. Sci. v.19, p.1623–1631. Doi: 10.5713/ajas.2006.1623 |
| 473 | Haddad, S.G., K.I. Ereifej. 2004. Substituting Bread By-product for Barley Grain in Fattening Diets for Baladi Kids. Asian-australas. J. Anim. Sci. v.17, p.629–632. Doi: 10.5713/ajas.2004.629 |
| 474 | Patra, A.K., K. Sharma, N. Dutta, A.K. Pattanaik. 2006. Effects of partial replacement of dietary protein by a leaf meal mixture on nutrient utilization by goats in pre- and late gestation. Small Rumin. Res. v.63, p.66-74. Doi: 10.1016/j.smallrumres.2005.02.008 |
| 475 | El Hag, M.G., M.A. Al-Merza, B. Al Salti. 2002. Growth in the Sultanate of Oman of Small Ruminants Given Date Byproducts-Urea Multinutrient Blocks. Asian-australas. J. Anim. Sci. v.15, p.671–674. Doi: 10.5713/ajas.2002.671 |
| 476 | Aregheore, E.M. 2001. Nutritive Value and Utilization of Three Grass Species by Crossbred Anglo-Nubian Goats in Samoa. Asian-australas. J. Anim. Sci. v.14, p.1389–1393. Doi: 10.5713/ajas.2001.1389 |
| 477 | Shahjalal, M., J.H. Topps. 2000. Feeding Sesbania Leaves as a Sole Feed on Growth and Nutrient Utilization in Goats. Asian-australas. J. Anim. Sci. v.13, p.487–489. Doi: 10.5713/ajas.2000.487 |
| 479 | Dahlan, I., M. Islam, M.A. Rajion. 2000. Nutrient Intake and Digestibility of Fresh, Ensiled and Pelleted Oil Palm (Elaeis guineensis) Frond by Goats. Asian-australas. J. Anim. Sci. v.13, p.1407–1413. Doi: 10.5713/ajas.2000.1407 |
| 480 | Dutta, N.; K. Sharma, Q.Z. Hasan. 1999. Effect of supplementation of rice straw with Leucaena leucocephala and Prosopis cineraria leaves on nutrient utilization by goats. Asian-australas. J. Anim. Sci. v.13, p.742–746. Doi: 10.5713/ajas.1999.742 |
| 481 | Srivastava, S.N.L., K. Sharma. 1998. Feed intake, nutrient utilization and growth rate of Jamunapari goats fed sundried Leucaena leucocephala. Asian-australas. J. Anim. Sci. v.13, p.742–746. Doi: 10.5713/ajas.1998.337 |
| 482 | El Hag, M.G., K.M. El Hargi. 1996. Feedlot performance and carcass characteristics of local (Dhofari) and exotic (cashmere) goats fed on a high-fiber by-products diet supplemented with fish sardine. Asian-australas. J. Anim. Sci. v.9, p.389–396. Doi: 10.5713/ajas.1996.389 |
| 483 | Huq, M.A., S. Akhter, M.A. Hashem, M.A.R. Howlider, M. Saadullah, M.M. Hossain. 1996. Growth and feed utilization in Black Bengal goats on road side grass-based diet supplement with fish meal and urea molasses block. Asian-australas. J. Anim. Sci. v.9, p.155–158. Doi: 10.5713/ajas.1996.155 |
| 484 | Ismartoyo, R. M. Dixon, R. F. Slocornbe, J. H. G. Holmes. 1993. Lablab purpureus seed as a supplement for goats fed low quality roughage. Asian-australas. J. Anim. Sci. v.6, p.515–519. Doi: 10.5713/ajas.1993.515 |
| 489 | Fernandes, M.H.M.R., K.T. Resende, L.O. Tedeschi, J.S. Fernandes, Jr., H.M. Silva, G.E. Carstens, T.T. Berchielli, I.A.M.A. Teixeira, L. Akinaga. 2007. Energy and protein requirements for maintenance and growth of Boer crossbred kids. J. Anim. Sci. v.85, p.1014-1023. Doi: 10.2527/jas.2006-110 |
| 491 | Moore, J.A., M.H. Poore, J.M. Luginbuhl. 2002. By-product feeds for meat goats: effects on digestibility, ruminal environment, and carcass characteristics. J. Anim. Sci. v.80, p.1752-1758. Doi: 10.2527/2002.8071752x |
| 492 | Luginbuhl, J.M., M.H. Poore, A.P. Conrad. 2000. Effect of level of whole cottonseed on intake, digestibility, and performance of growing male goats fed hay-based diets. J. Anim. Sci. v.78, p.1677-1683. Doi: 10.2527/2000.7861677x |
| 497 | Pailan, G.H., S.K. Mahanta, N.C. Verma, S.S. Kundu. 2007. Performance of sheep and goats maintained on rotational grazing with different levels of concentrate supplementation. Indian J. Anim. Sci. v.77, p.1161-1165. |
| 498 | Sankhayan, S.K., A.K. Shinde, B. Raghavendra, S.A. Karim. 2002. Effect of concentrate supplementation on growth performance and slaughter characteristics of weaner kids under silivipastoral grazing management. Indian J. Anim. Sci. v.72, p.101-103. |
| 499 | Ananda, S., V.R.B. Sastry, Agrawal, D.K. 1996. Effect of feeding urea-ammoniated neem (Azadirachta indica) seed kernel-meal on the performance of growing kids. Indian J. Anim. Sci. v.66, p.1285-1290. |
| 501 | Tamang, Y., G. Samanta. 1993. Feeding value of azolla (Azolla pinnata) an aquatic fern in Black Bengal goats. Indian J. Anim. Sci. v.63, p.188-191. |
| 503 | Kishan, J., M.Y. Khan, R.S. Dass, D.C. Joshi. 1986. Nutritional evaluation of subabul leucaena leucocephala in the ration of growing goats. Indian J. Anim. Sci. v.56, p.805-808. |
| 504 | Thakur, S., J.P. Srivastava, A.K. Verma, B.S. Gupta. 1982. Note on the utilization of poultry excreta as a protein source in diets of growing kids. Indian J. Anim. Sci. v.52, p.1260-1262. |
| 505 | Adebowale, E.A. 1982. Growth, economics and carcass characteristics of goats fed maize cobs (Zea mays Linn.), yam peels (Dioscorea cayenensis Lam.) and plantain peels (Musa paradisiaca Linn.). Indian J. Anim. Sci. v.52, p. 288-292. |
| 506 | Singhal, K.K.; V.D. Mudgal. 1981. Comparative study of urea and biuret on nutrient utilization and growth in goats. Indian J. Anim. Sci. v.51, p. 55-60. |
| 507 | Singh, C., P. Kumar, A. Rekib, D.K. Bhutani, 1980. Influence of supplementation of barley grain to green berseem diets of barbari kids. Indian J. Anim. Sci. v.50, p.857-860. |
| 509 | Yagoub Y.M., S.A. Babiker. 2008. Effect of dietary energy level on growth and carcass characteristics of female goats in Sudan. Livest. Res. Rural. Dev. v.20. |
| 513 | Titi, H.H., Dmour, R.O., Abdullah, A.Y. 2008. Growth performance and carcass characteristics of Awassi lambs and Shami goat kids fed yeast culture in their finishing diet. Anim. Feed Sci. Technol. v.142, p.33–43. Doi: 10.1016/j.anifeedsci.2007.06.034 |
| 514 | Soodeen-Karamath, S. F.G. Youssef. 1999. Effect of monensin, avoparcin and grass supplementation on utilization of urea-treated rice straw by sheep and goats. Small Rumin. Res. v.33, p.201-211. Doi: 10.1016/S0921-4488(99)00031-0 |
| 515 | Aregheore, E.M. 2000. Chemical composition and nutritive value of some tropical by-product feedstuffs for small ruminants - in vivo and in vitro digestibility. Anim. Feed Sci. Technol. v.85, p.99–109. Doi: 10.1016/S0377-8401(00)00123-1 |
| 516 | García, E.M., A. López, M. Zimerman, O. Hernández, J.I. Arroquy, M.A. Nazareno. 2019. Enhanced oxidative stability of meat by including tannin-rich leaves of woody plants in goat diet. Asian-australas. J. Anim. Sci. v.32, p.1439–1447. Doi: 10.5713/ajas.18.0537 |
| 517 | Medeiros, A.N., K.T. Resende, I.A.M.A. Teixeira, M.J. Araújo, E.A. Yáñez, A.C.D. Ferreira. 2014. Energy requirements for maintenance and growth of male saanen goat kids. Asian-australas. J. Anim. Sci. v.27, p.1293–1302. Doi: 10.5713/ajas.2013.13766 |
| 518 | Dos Santos, A.C.S., S.A. Santos, G.G.P. Carvalho, L.D.S. Mariz, M.SL. Tosto, S.C. Valadares Filho, J.A.G. Azevedo. 2018. A comparative study on the excretion of urinary metabolites in goats and sheep to evaluate spot sampling applied to protein nutrition trials. J. Anim. Sci. v.96, p. 3381-3397. Doi: 10.1093/jas/sky198 |
| 524 | Dida, M.F., Challi, D.G, Gangasahay, K.Y. 2019. Effect of feeding different proportions of pigeon pea (Cajanus cajan) and neem (Azadirachta indica) leaves on feed intake, digestibility, body weight gain and carcass characteristics of goats. Vet. Anim. Sci. v.8, p.100079. Doi: 10.1016/j.vas.2019.100079 |
| 525 | Hidosa, D., A. Tolera, A. Nurfeta. 2018. Effect of lablab and pigeon pea leaf meal supplementation on performance of goats fed a basal diet of haricot bean haulms. Trop. Anim. Health Prod. v.50, p.1271-1277. Doi: 10.1007/s11250-018-1554-2 |
| 526 | Yisehak, K., Y. Kibreab, T. Taye, M.R.A. Lourenço, G.P.J. Janssens. 2016. Response to dietary tannin challenges in view of the browser/grazer dichotomy in an Ethiopian setting: Bonga sheep versus Kaffa goats. Trop. Anim. Health Prod. v.48, p.125-131. Doi: 10.1007/s11250-015-0931-3 |
| 535 | Roy, A., G.P. Mandal, A.K. Patra. 2013. Evaluating the performance, carcass traits and conjugated linoleic acid content in muscle and adipose tissues of Black Bengal goats fed soybean oil and sunflower oil. Anim. Feed Sci. Technol. v.185, p.43-52. Doi: 10.1016/j.anifeedsci.2013.07.004 |
| 539 | El-Meccawi, S., M. Kam, A. Brosh, A.A. Degen. 2008. Heat production and energy balance of sheep and goats fed sole diets of Acacia saligna and Medicago sativa. Small Rumin. Res. v.75, p.199-203. Doi: 10.1016/j.smallrumres.2007.10.005 |
| 541 | Haddad, S.G., B.S. Obeidat. 2007. Production efficiency and feeding behavior of Awassi lambs and Baladi kids fed on a high concentrate diet. Small Rumin. Res. v.69, p.23-27. Doi: 10.1016/j.smallrumres.2005.12.004 |
| 543 | Candyrine, S.C.L., M.F. Jahromi, M. Ebrahimi, W.L. Chen, S. Rezaei, Y.M. Goh, N. Abdullah, J.B. Liang. 2019. Oil supplementation improved growth and diet digestibility in goats and sheep fed fattening diet. Asian-Australas. J. Anim. Sci. v.32, p.533-540. Doi: 10.5713/ajas.18.0059 |
| 545 | Abegunde, T.O., A.O. Akinsoyinu. 2011. Replacement effects of Panicum maximum with Ficus polita on performance of West African dwarf goats. J. Anim. Physiol. Anim. Nutr. v.95, p.192-197. Doi: 10.1111/j.1439-0396.2010.01040.x |
| 554 | El Hag, M.G., O.I. Kurdi, S.O. Mahgoub. 1985. Performance and carcass characteristics of Sudan desert sheep and goats on high roughage diets with added fat. Anim. Feed Sci. Technol. v.13, p.147-153. Doi: 10.1016/0377-8401(85)90050-1 |
| 561 | Asmare, A., R. Puchala, K. Tesfai, G.D. Detweiler, L.J. Dawson, A.R. Askar, T. Sahlu, Z. Wang, A.L. Goetsch. 2011. Effects of small ruminant type and restricted protein intake on metabolism. Small Rumin. Res. v.98, p.111-114. Doi: 10.1016/j.smallrumres.2011.03.027 |
| 562 | Carmichael, A.K., B. Kouakou, S. Gelaye, G. Kannan, J.H. Lee, T.H. Terrill. 2012. Organ mass and composition in growing dairy goat wethers fed different levels of poultry fat and protein. Small Rumin. Res. v.104, p.104-113. Doi: 10.1016/j.smallrumres.2011.09.046 |
| 4052 | Odeyinka, S.M., O.J. Oyedele, P.A. Olubunmi. 2003. The performance of West African Dwarf goats on soybean milk residue, cowpea seed waste and corn starch residue. Livest. Res. Rural. Dev. v.15. |
| 4062 | Phengsavanh, P., I. Ledin. 2003. Effect of Stylo 184 (Stylosanthes guianensis CIAT 184) and Gamba grass (Andropogon gayanus cv. Kent) in diets for growing goats. Livest. Res. Rural. Dev. v.15. |

^1^Publication ID refers to the publication code for each study in the database.

**Table S2.** Mean comparison of potential variables affecting energy requirements in sheep and goats raised in contrasting climate regions.

| Variables^1^ | Climate region | | | | | P-value |
| --- | --- | --- | --- | --- | --- | --- |
|  | Tropical | Subtropical | Arid | Semi-arid | Mediterranean |  |
| ME intake, MJ/BW^0.75^ | 0.71 ± 0.02^ab^ | 0.64 ± 0.04^a^ | 0.80 ± 0.04^bc^ | 0.83 ± 0.03^c^ | 0.94 ± 0.04^c^ | <0.01 |
| CP, % DM | 14.7 ± 0.46 | 14.9 ± 0.83 | 13.5 ± 1.00 | 13.9 ± 0.66 | 15.1 ± 0.80 | 0.62 |
| NDF, % DM | 49.6 ± 1.44^b^ | 49.3 ± 3.29^b^ | 46.0 ± 3.40^b^ | 43.5 ± 2.37^b^ | 32.2 ± 2.53^a^ | <0.01 |
| DMD, % DM | 63.4 ± 0.86^a^ | 61.4 ± 1.49^a^ | 64.0 ± 1.82^ab^ | 62.8 ± 1.26^a^ | 68.9 ± 1.57^b^ | 0.01 |
| OMD, % DM | 64.2 ± 0.87^a^ | 63.9 ± 1.47^a^ | 70.9 ± 2.43^ab^ | 65.9 ± 1.25^ab^ | 70.7 ± 1.25^b^ | <0.01 |
| NDFD, % DM | 59.0 ± 1.13 | 52.7 ± 2.09 | 58.6 ± 3.09 | 54.8 ± 1.91 | 56.3 ± 1.89 | 0.06 |
| SRW, kg | 43.7 ± 1.91^a^ | 51.0 ± 3.43^ab^ | 53.7 ± 3.90^ab^ | 49.9 ± 2.73^ab^ | 57.2 ± 3.43^b^ | <0.01 |
| Z | 0.47 ± 0.02^ab^ | 0.39 ± 0.03^a^ | 0.55 ± 0.04^bc^ | 0.53 ± 0.02^bc^ | 0.59 ± 0.03^c^ | <0.01 |
| *n* | 477 | 124 | 93 | 176 | 122 |  |

^1^ME: metabolizable energy, Z: degree of maturity given by the ratio of current weight /standard reference weight at maturity (CSIRO, 2007); CP: crude protein content; NDF: neutral detergent fiber; DMD: dry matter digestibility; NDFD: neutral detergent fiber digestibility; SRW: standard reference weight; OMD: organic matter digestibility; *n*: number of treatment means.

**Table S3.** Prediction equations of metabolizable energy requirements (MJ/kg BW^0.75^) using average daily gain (g/kg BW^0.75^) of sheep from different sexes.

| Item | Intact Male^1^ | Female^2^ | Castrated Male^3^ |
| --- | --- | --- | --- |
| Model n. | S1 | S2 | S3 |
| **Intercept** | 0.478 ± 0.025 (<0.01) | 0.687 ± 0.115 (<0.01) | 0.550 ± 0.078 (<0.01) |
| **Slope [ADG (g/kg BW^0.75^)]** | 0.031 ± 0.002 (<0.01) | 0.013 ± 0.010 (0.24) | 0.022 ± 0.004 (<0.01) |
| *n* | 488 | 26 | 35 |
| *Monte Carlo cross-evaluation^1^* |  |  |  |
| RMSE | 0.18 | 0.21 | 0.23 |
| RMSPE | 23.3 | 28.8 | 30.7 |
| RSR | 0.74 | 1.80 | 1.47 |
| *Mean bias* | 2.86 | 41.3 | 37.3 |
| *Slope bias* | 1.92 | 24.0 | 33.4 |
| CCC | 0.64 | 0.13 | 0.33 |

^1^Root mean square error (RMSE), root mean square percentage error (RMSPE), RMSPE-observations standard deviation ratio (RSR), and concordance correlation coefficient (CCC).

**Table S4.** Prediction equations of metabolizable energy requirements (MJ/kg BW^0.75^) using average daily gain (g/kg BW^0.75^) of goats from different sexes.

| Item | Intact Male^1^ | Female^2^ | Castrated Male^3^ |
| --- | --- | --- | --- |
| Model n. | S4 | S5 | S6 |
| **Intercept** | 0.511 ± 0.031 (<0.01) | 0.702 ± 0.124 (<0.01) | 0.534 ± 0.059 (<0.01) |
| **Slope [ADG (g/kg BW^0.75^)]** | 0.028 ± 0.003 (<0.01) | 0.014 ± 0.010 (0.04) | 0.019 ± 0.005 (<0.01) |
| *n* | 161 | 29 | 52 |
| *Monte Carlo cross-evaluation^1^* |  |  |  |
| RMSE | 0.19 | 0.41 | 0.20 |
| RMSPE | 26.6 | 60.2 | 31.5 |
| RSR | 0.93 | 1.45 | 1.20 |
| *Mean bias* | 9.03 | 31.5 | 29.8 |
| *Slope bias* | 8.38 | 15.4 | 21.5 |
| CCC | 0.43 | 0.01 | 0.23 |

^1^Root mean square error (RMSE), root mean square percentage error (RMSPE), RMSPE-observations standard deviation ratio (RSR), and concordance correlation coefficient (CCC).

## Screening

## Included

## Eligibility

## Identification

Records screened
(*n*=295)

Total of studies collected from online database search

(*n*=24)

Studies included in

meta-analysis (n = 37)

n = 39)

Records removed by advanced search of title/abstract
(*n*=582)

Records identified through database searching
(*n*=844)

Full-text articles assessed for eligibility
(*n*=179)

Full-text articles excluded, with reasons (*n*=155):

-Review articles (*n*=21)

-Studies with only abstracts available (*n*=12)

-Studies without BW records (*n*=48)

-Studies that provided insufficient information such as dietary composition to allow metabolizable energy intake estimation or have not reported it (*n*=74)

International collaboration with H. Archimede et al. (INRAE)

(*n*=151)

Total of studies included in the meta-analysis (*n*=175)

**Figure S1.** Scheme of the search and selection process for sheep studies (PRISMA) (Liberati et al., 2009). Key words: sheep, feeding trial, requirement, and tropics; in title and abstract; Date: from 2013 to 2019.

## Screening

## Included

## Eligibility

## Identification

Records screened
(*n*=147)

Total of studies collected from online database search

(*n*=8)

Studies included in

meta-analysis (n = 37)

n = 39)

Records removed by advanced search of title/abstract
(*n*=234)

Records identified through database searching
(*n*= 381)

Full-text articles assessed for eligibility
(*n*=57)

Full-text articles excluded, with reasons (*n*=49):

-Review articles (*n*=2)

-Studies without BW records (*n*=7)

-Studies that provided insufficient information such as dietary composition to allow metabolizable energy intake estimation or have not reported it (*n*=41)

International collaboration with H. Archimede et al. (INRAE)

(*n*=93)

Total of studies included in the meta-analysis

(*n*=101)

**Figure S2.** Scheme of the search and selection process for goat studies (PRISMA) (Liberati et al., 2009). Key words: goat, feeding trial, requirement, and tropics; Date: from 2013 to 2019.


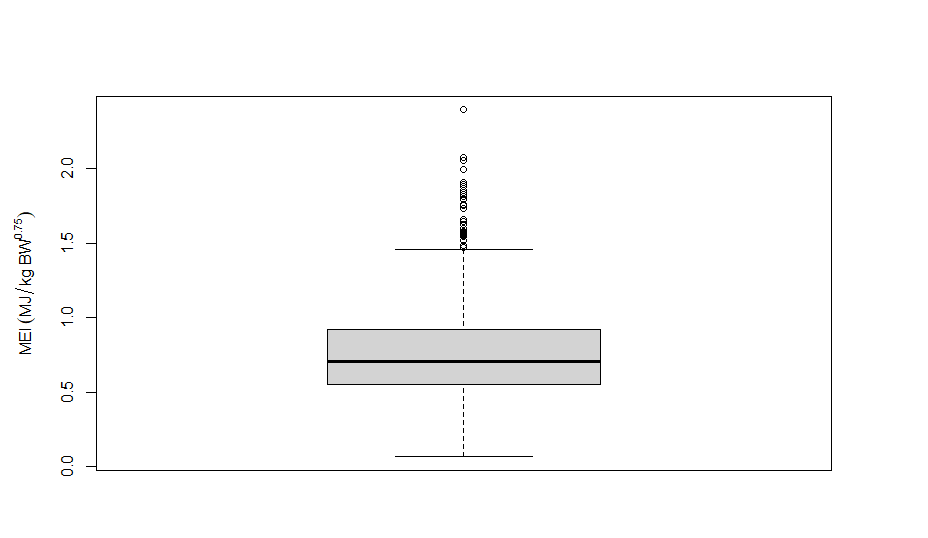


**Figure S3.** Boxplot for outlier identification of metabolizable energy intake (MEI; MJ/kg^0.75^).


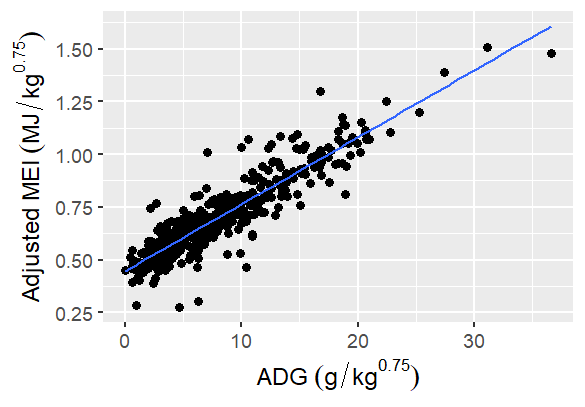


**Figure S4.** Relationship between metabolizable energy intake (MEI) and average daily gain (ADG) for the tropical model (model 9). Metabolizable energy intake observations were adjusted for the random study effect (St-Pierre, 2001).


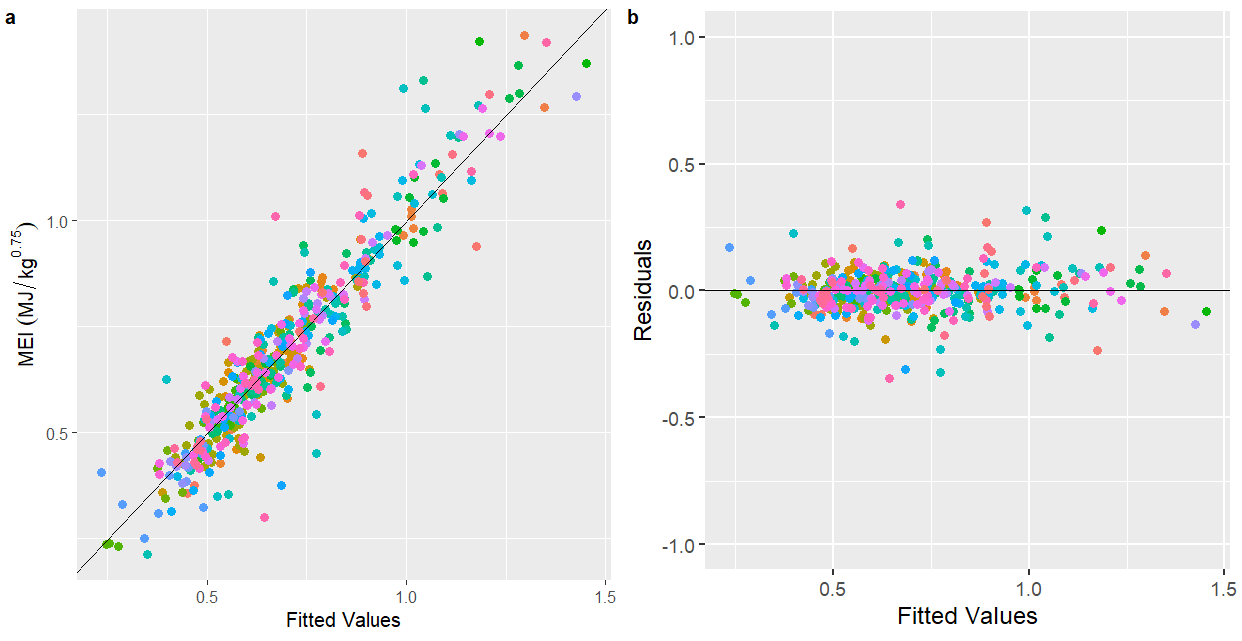


**Figure S5.** Observed versus predicted (panel a) and residuals versus predicted (panel b) values for metabolizable energy intake (MJ/kg^0.75^) for the tropical model (model 9). Each point represents a treatment mean (n = 477), and studies are represented by color.

**References**

CSIRO. 2007. Nutrient requirements of domesticated ruminants. CSIRO Publishing, Melbourne.

St-Pierre, N. R. 2001. Invited review: Integrating quantitative findings from multiple studies using mixed model methodology. J Dairy Sci 84(4):741-755. doi: 10.3168/jds.S0022-0302(01)74530-4
